# Dexamethasone enhances the efficacy of atorvastatin in inhibiting excessively inflammation-induced abnormal angiogenesis by regulating macrophages

**DOI:** 10.1186/s12974-021-02257-1

**Published:** 2021-09-15

**Authors:** Zhitao Gong, Daqiang Zhan, Meng Nie, Xiaochun Li, Chuang Gao, Xuanhui Liu, Tangtang Xiang, Jiangyuan Yuan, Weiwei Jiang, Jinhao Huang, Wei Quan, Dong Wang, Ye Tian, Hengjie Yuan, Jianning Zhang, Rongcai Jiang

**Affiliations:** 1 Department of Neurosurgery, Tianjin Medical University General Hospital, Tianjin Medical University, Tianjin, 300052 China; 2 Tianjin Neurological Institute, Key Laboratory of Post-neuroinjury Neuro-repair and Regeneration in Central Nervous System, Ministry of Education and Tianjin, Tianjin, China; 3 Department of Rehabilitation Medicine, The Second Affiliated Hospital of Anhui Medical University, Hefei, China; 4 Department of Pharmacy, Tianjin Medical University General Hospital, Tianjin Medical University, Tianjin, China; 5 Department of pharmacy, Second Affiliated Hospital of Hainan Medical University, Hainan, China

**Keywords:** Chronic subdural haematoma, Atorvastatin, Dexamethasone, Macrophages, Inflammation, Angiogenesis

## Abstract

**Background:**

We have recently showed that atorvastatin (ATO) combined with low dose of dexamethasone (DEX) was more efficacious in treating patients with chronic subdural haematoma (CSDH) than ATO monotherapy. This study was designed to investigate the underlying mechanisms of the improved efficacy of this combined therapy.

**Methods:**

Mass spectrometry was performed to quantitatively detect drugs in haematoma fluids and serum samples from CSDH patients and also in cultured macrophages after treatment with either ATO alone or in combination with DEX. The differentiation and apoptosis of macrophages were evaluated using flow cytometry. The expression of cytokines, chemokines and angiogenesis-related proteins was evaluated using proteome profile arrays, immunoblots and ELISA, respectively.

**Results:**

ATO was detected in haematoma fluids and serum samples, whose levels were increased significantly in samples collected from patients treated with both ATO and DEX. ATO was also increased in cultured macrophages treated with ATO and DEX. The numbers of M1-polarized macrophages were higher than the M2 phenotype in the haematoma fluids of patients. Cultured macrophages treated with ATO and DEX had reduced numbers of M1-polarized macrophages, increased numbers of M2-polarized macrophages as compared to monotherapies, and decreased rate of apoptosis induced by high-dose DEX. DEX enhanced the anti-inflammatory and anti-angiogenic activity of ATO by suppressing VEGFA and other inflammatory angiogenic factors. Consistent with the finding, patients responded well to the drug treatments had lower serum levels of VEGFA.

**Conclusions:**

We have shown for the first time that ATO given orally was detected in CSDH haematoma fluids. DEX enhances the anti-inflammatory and anti-angiogenic effects of ATO, primarily by increasing the presence of ATO in haematoma and macrophages and by regulating the functions of macrophages.

**Supplementary Information:**

The online version contains supplementary material available at 10.1186/s12974-021-02257-1.

## Background

Chronic subdural haematoma (CSDH) often develops after mild to moderate head trauma and is formed by progressive accumulation of blood in a confined capsule surrounded by membrane with extensive, but aberrant angiogenesis. Increasing evidence suggests that CSDH is formed through interconnected pathways of inflammation, aberrant angiogenesis, coagulopathy, dysfunctional fibrinolysis, recurrent microbleeds, exudation of plasma components and release of cytokines [1–5]. The molecular pathophysiology underlying these processes is not fully understood. While surgery is safe and the first line treatment of CSDH, it results in recurrent CSDH in 5–25% of patients [6, 7]. Because CSDH mostly occurs in the elderly adults, and many of them have comorbidities such as cardiopulmonary diseases, surgery may be contraindicated or carries higher risk of complications [8–10]. Safe and effective non-surgical treatments targeting the above-mentioned pathologies are needed to improve the outcome of CSDH patients.

Angiotensin-converting enzyme inhibitors, COX-2 inhibitors, tranexamic acid, and platelet-activating factor receptor antagonists have been examined as alternatives to surgery for patients with CSDH [11–13]. However, these drugs have either failed in randomized clinical trials or have not been tested in such trials [6, 12]. Dexamethasone (DEX) has been shown to resolve CSDH and reduce its recurrence at high dosages [14], but its benefits are often offset by serious adverse effects such as hyperglycaemia, infections, and mental changes [10, 11, 15]. We have shown that atorvastatin (ATO) reduces haematoma volumes, improves the outcomes of patients with CSDH, and reduces the need for surgery [16, 17]. However, the efficacy of ATO varies considerably among patients receiving the treatment [18, 19] and approximately 11.2% of CSDH patients fail to respond to ATO [20]. Moreover, ATO monotherapy lasts more than 8 weeks to achieve the optimal outcome. We have recently developed a new treatment regimen of combining ATO with low-dose DEX to improve the efficacy of ATO without serious side effects associated with the high dose of DEX [20]. Here, we report results from a study designed to investigate the molecular mechanisms of the combined effects of ATO and DEX.

## Methods

### Patients and treatments

All patients provided written consent to the study and received careful clinical and neurosurgical assessments before being enrolled into the trial, as we described previously [16, 20]. The inclusion and exclusion criteria are listed in the Additional files section. Briefly, patients with mild or moderate CSDH who had a minimal risk of cerebral hernia and did not need immediate surgery were included in the trial. They were randomized to receive ATO monotherapy (20 mg daily) or a combined regimen of ATO 20 mg daily and DEX for five weeks. DEX was given at 2.25 mg daily for 2 weeks followed by 1.5 mg daily for 2 weeks and then 0.75 mg daily for 1 week [20]. Patients were switched to surgery to remove haematomas when their neurological deficits deteriorated or when CT or MRI scan found haematoma enlargement and/or a midline shift of more than 1 cm.

During the trial, peripheral venous blood samples were collected at the baseline before treatment, 7 days and 5 weeks after the treatment started. Haematoma fluids were collected during surgery from patients who underwent surgery without the drug treatment and those who were switched to surgery after unsuccessful drug treatment. Blood samples were collected from these patients immediately before and 7 days after surgery. Venous blood samples were drawn into a serum separator tube and left to stand at room temperature for 30 min to ensure full coagulation to collect serum. CSDH fluids were collected into vacuum tubes and centrifuged at 2000*g* for 20 min at 4 °C to collect the supernatants. Both haematoma supernatants and serum samples were aliquoted and stored at −80 °C until analyses [21]. Cells in haematoma freshly collected from CSDH were immediately analyzed using flow cytometry.

### Cell culture and drug treatments

It has been shown that haematoma fluid contains high levels of angiogenic factors than in blood, including vascular endothelial growth factor (VEGFA), IL-1β, IL-6 and IL-8. These factors are primarily produced by monocytes/macrophages and contribute to inflammation-induced formation and aberrant angiogenesis of the CSDH membrane [4, 22, 23]. In particular, VEGFA produced by infiltrating macrophages in the haematoma capsule is thought to play a major role in inducing ongoing rebleeding [24, 25]. This is because VEGFA is the most important proangiogenic factor involved in excessive microvascular permeability [9, 26, 27]. Macrophage-derived VEGFA or other angiogenic factors were one of major focuses of the study.

Cells from the human acute monocytic leukaemia cell line (THP-1; 3111C0001CCC000057, NICR, China), which a well-established monocytic cell line phenotypically homologous to primary human monocytes, can be differentiated into macrophages by phorbol-12-myristate-13-acetate (PMA) [28]. They were seeded in 6-well plates at a density of 1×10^6^/well and cultured in the RPMI 1640 medium (C11875500BT, Gibco, New Zealand) supplemented with 10% fetal bovine serum (10091-148, Gibco, New Zealand) in a humidified 5% CO2 incubator. After stimulation with 0.1 μM PMA (Sigma-Aldrich, St Louis, MO) for 24 h, the cells were washed and cultured in the regulator growth medium for 24 h at 37 °C with the following treatments: (1) untreated control; (2) 100 μg/L lipopolysaccharide (LPS; Sigma-Aldrich); (3) LPS and 10 μM ATO (Pfizer, USA); (4) LPS and 0.1 μM ATO; (5) LPS and 1 μM DEX (Sigma-Aldrich); (6) LPS and 0.01 μM DEX; and (7) LPS and a combination of 0.1 μM ATO and 0.01 μM DEX. The treated cells and conditioned media were harvested for analyses.

### Mass spectrometry (MS)

Serum samples and haematoma fluids were extracted with ethyl acetate, and cell samples were processed with acetonitrile for protein precipitation before LC-MS/MS. Ultra-high-performance liquid chromatography (UPLC) coupled with triple quadrupole MS was performed to detect ATO, its two active metabolites ortho-hydroxy-atorvastatin (o-ATO) and para-hydroxy-atorvastatin (p-ATO), and DEX in haematoma fluids, serum samples, and cultured cells. A Waters ACQUITY UPLC I-Class system (Waters, USA) equipped with an Acquity UPLC BEH C18 column (1.7 μm, 100×2.1 mm, Waters, USA) was coupled online to a Waters Xevo TQD IVD triple quadrupole mass spectrometer (Waters, Ireland) with electrospray ionization and selective reaction monitoring in positive ion mode. Solvent A (acetonitrile with 0.01% formic acid) and solvent B (0.01% aqueous formic acid) were used with a flow rate of 0.4 mL/min and the following solvent (time [min], vol% solvent B): 0.0, 80%; 1.0, 60%; 4, 10%; 4.5, 90%; 4.51, 80%; and 5, 80%. The injection volume was 10 μL.

Drugs in cells, including those binding to the cell membrane, were extracted in a lysis buffer. Some cells were treated with 0.25% trypsin-EDTA (25200-056, Gibco, New Zealand) to remove the surface-bound drugs before being lysed.

### Flow cytometry

Cells were trypsinized and collected by centrifugation at 800 rpm for 5 min and washed with ice-cold PBS. They were double stained for 30 min at 4 °C with either fluorochrome-tagged antibodies against CD11b-421 (1: 1000, BioLegend, San Diego, CA, USA) and CD86-PE (1:1000, BioLegend) to identify the proinflammatory macrophages (M1) or the CD11b-421 antibody and CD163-PE/Cy7 (1:1000, BioLegend) to identify the anti-inflammatory macrophages (M2). After washing with PBS to remove excess antibodies, cells were analyzed using flow cytometry (LSRFortessa^TM^ Cell Analyzer, Cat No. 647780P3 BD) and FlowJo version 10 (BD, San Jose, USA). Macrophage apoptosis was determined by the binding of APC annexin V (Apoptosis Detection Kit with 7-AAD, BioLegend) according to the manufacturer’s instructions. Briefly, 5×10^5^ cells were incubated in 100 μL annexin V binding buffer containing 5 μL APC annexin V and 5 μL 7-AAD viability staining solution for 15 min at room temperature in the dark.

### Cytokine and angiogenesis arrays

Cytokines, chemokines and angiogenesis-related proteins secreted by macrophages into the conditioned media were detected using human cytokine (R&D Systems, Minneapolis, USA) and angiogenesis (R&D Systems, Minneapolis, USA) array kits and quantified by pixel density using Quantity One software version 4.6.6 (Bio-Rad, USA) [21].

### Immunoblots

Cultured macrophages were washed in ice-cold phosphate-buffered saline and lysed for 20 min on ice. The cell lysates were centrifuged at 13,000 rpm for 15 min at 4 °C to collect the supernatants. Total protein in the supernatant was quantified using the bicinchoninic acid assay (BCA1, Sigma-Aldrich). The volumes of cell lysates were standardized to an equal amount of protein (20 μg), mixed with 5X SDS sample containing 50 mM dithiothreitol, separated by 10% SDS-PAGE gels, and transferred onto nitrocellulose membranes. The membranes were blocked in 5% dried milk in TBS-T and 0.1% Tween 20, washed, and incubated with anti-IL-8 (ab18672, Abcam, Cambridge, UK, 0.1 μg/mL), anti-IL-1β (12242, CST, USA, 1:1000), anti-TGF-β1 (ab179695, Abcam, Cambridge, UK, 1:1000), anti-CD163 (ab182422, Abcam, Cambridge, UK, 1:1000), anti-CD206 (ab125028, Abcam, Cambridge, UK, 1:2000), anti-Scavenging Receptor SR-BI (ab217318, Abcam, Cambridge, UK, 1:2000), anti-Arginase-1 (9819, CST, USA, 1:1000), anti-OATP1A2 (ab221804, Abcam, Cambridge, UK, 1:1000) and β-actin (Sigma, Germany, 1:1000). After washing to remove excess antibodies, the membranes were incubated with appropriate secondary antibodies followed by chemiluminescence and fluorescence detection agent (Chemi XT4, Syngene, UK). Specific proteins were quantified by densitometry using the ImageJ analysis program (NIH, USA).

### ELISA

VEGFA, IL-6 and endothelin-1 (ET-1) were quantified using sandwich ELISA kits (R&D Systems) according to the manufacturer’s instructions. The optical density was measured with a microplate reader (5250040, Varioskan Flash, Thermo, USA) set to 450 nm, with the correction wavelength set to 540 nm.

### Quantitative real-time reverse transcriptase polymerase chain reaction (qRT-PCR)

mRNAs for the genes encoding P-glycoprotein, Cytochrome P450 3A4 (CYP3A4) and IL-10 in cultured macrophages were quantitatively amplified using qRT-PCR. RNA expression was normalized to GAPDH. Table S1 lists the primers used in this study.

### Statistical analysis

Categorical variables are presented as frequencies and percentages (no [%]) and were compared between groups using the Pearson chi-square test. Continuous variables are expressed as the mean ± standard deviation or median (25th–75th percentile), depending on a normal or skewed distribution of data determined by the Shapiro–Wilk test. Student’s *t* test, one-way analysis of variance (ANOVA) with Bonferroni correction, the Mann–Whitney *U* test or the Kruskal-Wallis H test were conducted according to the data distribution. Data were analyzed using SPSS version 22.0 (IBM, Chicago, USA), and a statistically significant level was defined as a *P* value of <0.05.

## Results

Haematoma fluids and serum samples from 24 patients who switched to surgery after unsuccessful drug treatment were collected (no serum samples obtained at the fifth week of medication before surgery because the median duration of medication before surgery was 8.5 days). For patients who responded well to drug treatments, serum samples were collected from 36 patients at the therapeutic time point designated for this study. Among the 60 patients whose clinical samples were successfully collected, 28 patients were receiving ATO monotherapy at the outset, and the remaining 32 patients receiving the combined therapy with ATO and DEX. The baseline characteristics did not differ significantly between patients receiving the ATO and those receiving ATO and DEX. However, there were fewer patients receiving the combined treatments were switched to surgery as compared to those receiving ATO monotherapy (28.13% vs. 53.57%, *P* = 0.045, Table S2). There was no significant difference in baseline characteristics between patients who responded well to the combined therapy and those who responded poorly and was switched to surgery, except for more male patients in surgery group (Table S3).

### ATO was detected in haematoma fluids and serum samples of CSDH patients receiving drug treatments, with higher levels in those receiving ATO and DEX

**Fig. 1:**
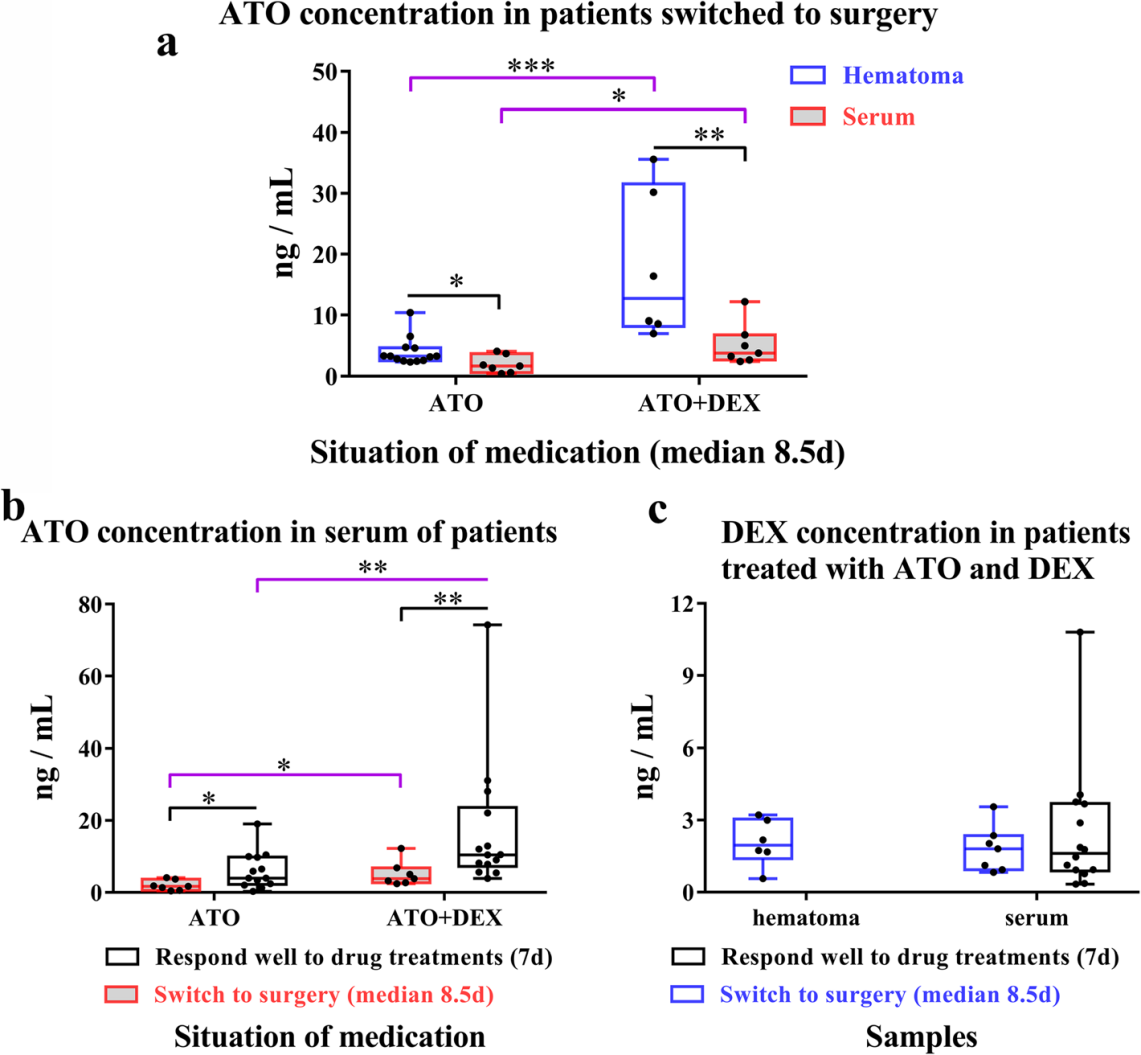
ATO and DEX in CSDH patients. **a** ATO concentration in haematoma fluids and serum samples of patients who were switched to surgery, ATO (haematoma vs. serum, Mann–Whitney *U* test, ^*^P = 0.03); ATO + DEX (haematoma vs. serum, Mann–Whitney *U* test, ^**^P = 0.008); haematoma (ATO vs. ATO + DEX, Mann–Whitney *U* test, ^***^P = 0.001); serum (ATO vs. ATO + DEX, Mann–Whitney *U* test, ^*^P = 0.026). **b** Serum ATO in patients who responded well to the drug treatments and those who switched to surgery, ATO (switch to surgery vs. respond well to drug treatments, Mann–Whitney *U* test, ^*^P = 0.046); ATO + DEX (switch to surgery vs. respond well to drug treatments, Mann–Whitney *U* test, ^**^P = 0.006); switch to surgery (ATO vs. ATO + DEX, Mann–Whitney *U* test, ^*^P = 0.026); respond well to drug treatments (ATO vs. ATO + DEX, Mann–Whitney *U* test, ^**^P = 0.007). **c** DEX concentration in patients who responded well to the drug treatments and those who switched to surgery. *Note*: ATO, atorvastatin monotherapy group; ATO + DEX, combination group treated with atorvastatin and low-dose dexamethasone

ATO, o-ATO, p-ATO and DEX were detected in haematoma fluids and serum samples of CSDH patients receiving the drug treatments. ATO in haematoma fluids were significantly higher than that in the serum samples of patients who were switched to surgery during the trial (*P* < 0.05, Fig. 1a). Additionally, ATO in haematoma fluids were higher in patients receiving ATO combined with DEX, as compared to those receiving ATO monotherapy (*P* = 0.001, Fig. 1a). Serum levels of ATO were higher in patients receiving combined therapy as compared to those receiving ATO alone, regardless if patients responded well to the drug treatment or were switched to surgery (Fig. 1a, b). Serum ATO in patients who responded well to the drug treatments was markedly higher than that in patients who switched to surgery after unsuccessful drug treatments (Fig. 1b). In contrast, there was no difference in serum level of DEX between patients who responded well to the drug treatment and those being switched to surgery. Levels of DEX in haematoma fluid and serum were comparable in patients who were switched to surgery. The concentrations of both o-ATO and p-ATO showed similar trends (Figure S1).

### ATO in macrophages was increased by DEX

**Fig. 2:**
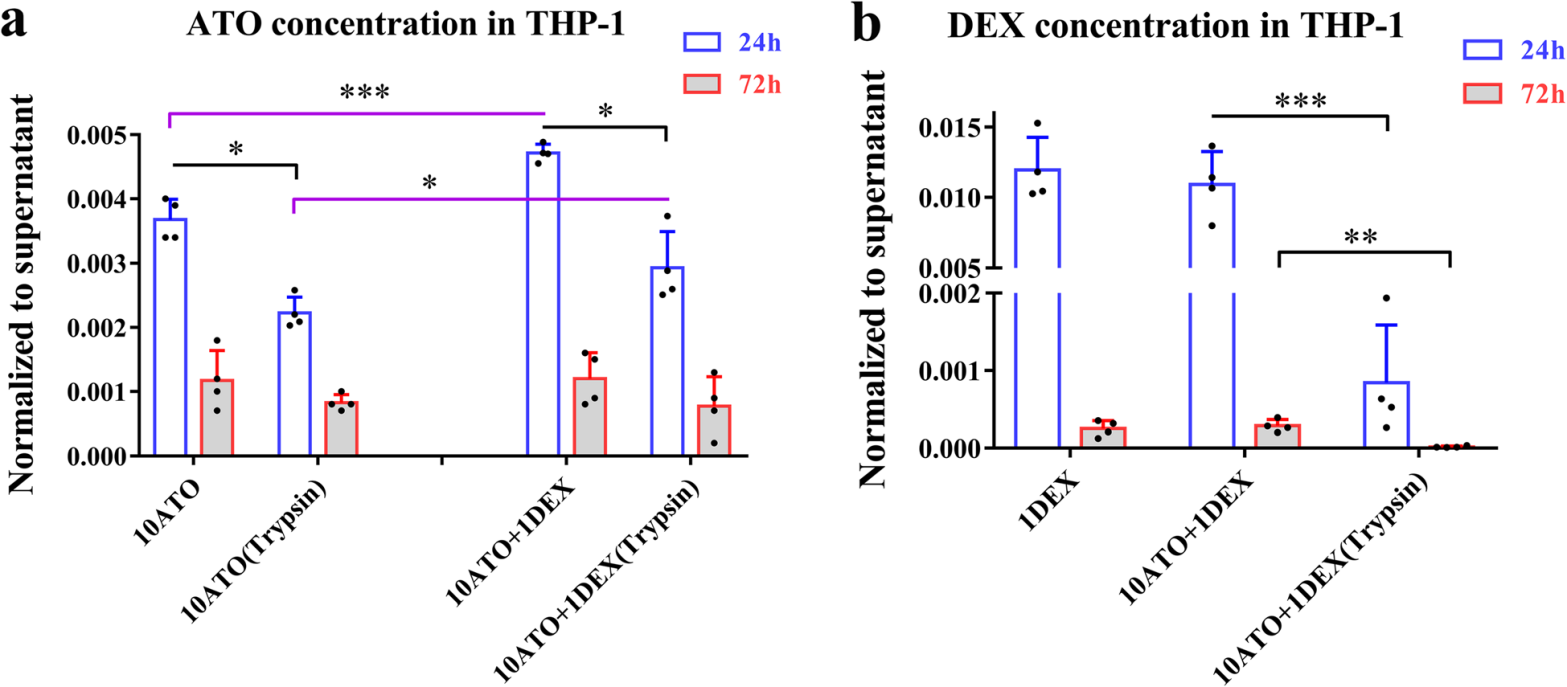
Concentrations of ATO and DEX in THP-1 macrophages. **a** ATO in macrophages, 10ATO—24 h vs. 10ATO (trypsin)—24 h, paired Student’s *t* test, ^*^P = 0.01; 10ATO + 1DEX—24 h vs. 10ATO + 1DEX (trypsin)—24 h, paired Student’s *t* test, ^*^P = 0.01; 10ATO—24 h vs. 10ATO + 1DEX—24 h, paired Student’s *t* test, ^***^P = 0.003; 10ATO (trypsin)—24 h vs. 10ATO + 1DEX (trypsin)—24 h, paired Student’s *t* test, ^*^P = 0.03. **b** The concentration of DEX in macrophages, 10ATO + 1DEX—24 h vs. 10ATO + 1DEX (trypsin)—24 h, paired Student’s *t* test, ^***^P = 0.004; 10ATO + 1DEX—72 h vs. 10ATO + 1DEX (trypsin)—72 h, paired Student’s *t* test, ^**^P = 0.009. NOTE: 10ATO, 10 μM atorvastatin; 10ATO+1DEX, 10 μM atorvastatin and 1 μM dexamethasone; 1DEX, 1 μM dexamethasone

ATO and DEX were measured in lysates of macrophages incubated with the drugs for 24 h before and after they were treated with trypsin. After trypsin treatment to remove surface-bound drugs, ATO decreased by 29.65 to 39.54% (Fig. 2a), whereas DEX reduced by 92.31 to 94.11% (Fig. 2b), suggesting that DEX was primarily membrane-bound, whereas ATO mostly entered into macrophages. ATO had a in macrophages showed a significantly greater level in macrophages treated with ATO and DEX, as compared to those treated with ATO alone (Fig. 2a). In contrast, levels of DEX remained comparable between cells treated with either ATO combined with DEX or DEX alone (Fig. 2b).

After 72 h in culture (the medium was replaced with drug-free media after 24 h with the drugs), DEX in macrophages and surface-bound ATO were not detected, with intracellular ATO being similar among cells receiving different treatments (Fig. 2). Both o-ATO and p-ATO were not detected in cultured macrophages. In addition, the total drug concentrations in human umbilical vein endothelial cells (HUVECs) were similar to that in macrophages, but showed no difference between cells treated with ATO and those with ATO combined with DEX (Figure S2).

In macrophages, the ATO efflux transporter P-glycoprotein [29] was markedly decreased after treatment with the combination regimen (*P* = 0.003), 1 μM DEX (*P* = 0.002) or 0.01 μM DEX (*P* = 0.012, Figure S3c). While not reaching statistical significance, the ATO uptake transporter organic anion-transporting polypeptide 1A2 (OATP1A2) [30] showed a trend for increase in macrophages treated with ATO and DEX (Figure S3a, b). The drug catabolism mediator CYP3A4 [31] increased after treatment with 10 μM ATO (*P* = 0.027) and also showed an upward trend after treatment with 0.1 μM ATO (*P* = 0.065). But the expression of CYP3A4 decreased after combined treatment with DEX (LPS+0.01D+0.1A group vs. LPS+0.1A group, *P* = 0.026, Figure S3d).

### DEX optimized the effect of ATO on macrophage differentiation

Macrophages, which are considered crucial for CSDH growth [4, 24, 26], were detected in haematoma fluids; among the CD45^+^/CD11b^+^/CD14^-^ macrophages, 36.47% were M1 macrophages and 8.66% were M2 macrophages (Figure S4). To further understand the underlying pharmacological mechanisms, subsets of cultured macrophages were identified by flow cytometry in vitro. PMA treatment increased M1 macrophages by 6.84-fold and increase M2 macrophages by 5.22-fold, without significant change of CD11b (Figure S5). LPS can effectively simulate the effect of haematoma on macrophages, while avoiding the extreme individual variations when macrophages were incubated with haematoma from different patients (Figure S6).

**Fig. 3:**
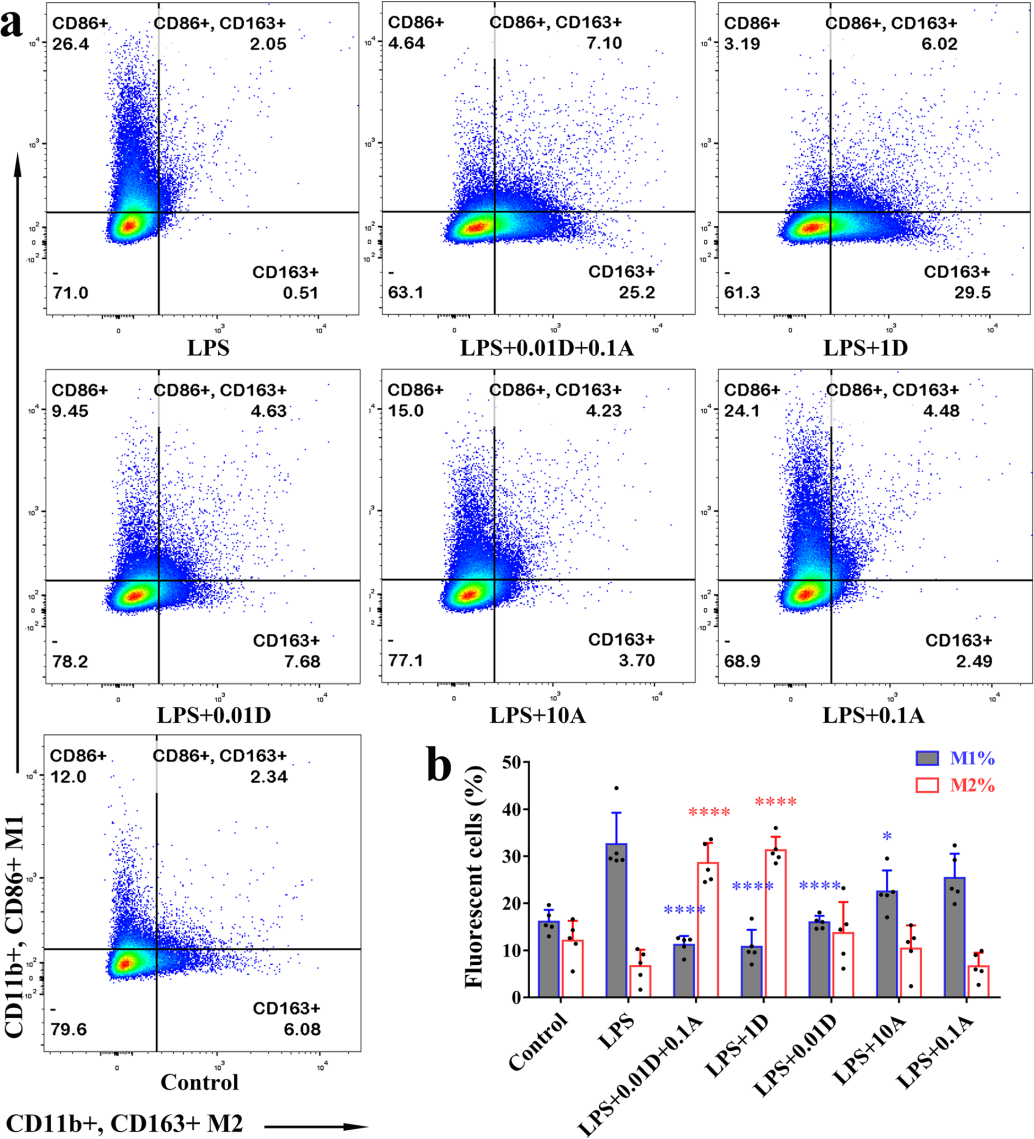
The effect of ATO and DEX on the differentiation of macrophages. **a** Representative images of differentiated macrophages defined by specific markers. **b** The differences of M1% among groups, one-way ANOVA, *P* < 0.001; compared with the LPS group, Bonferroni test, ^****^P < 0.001, ^*^P = 0.016. The differences of M2% among groups, one-way ANOVA, *P* < 0.001; compared with the LPS group, Bonferroni test, ^****^P < 0.001. *Note*: A, atorvastatin; D, dexamethasone

After treatment with the combination regimen (*P* < 0.001), 1 μM DEX (*P* < 0.001), 0.01 μM DEX (*P* < 0.001) or 10 μM ATO (*P* = 0.016), the percentage of M1 macrophages decreased compared with LPS group. But only the combination treatment and the treatment with 1 μM DEX increased the percentage of M2 macrophages (Fig. 3). The morphological changes of macrophages (Figure S7) and the median fluorescence intensity (MFI) changes of CD86 and CD163 (Figure S8) also suggest the combined treatment promotes the M2 polarization of macrophages.

**Fig. 4:**
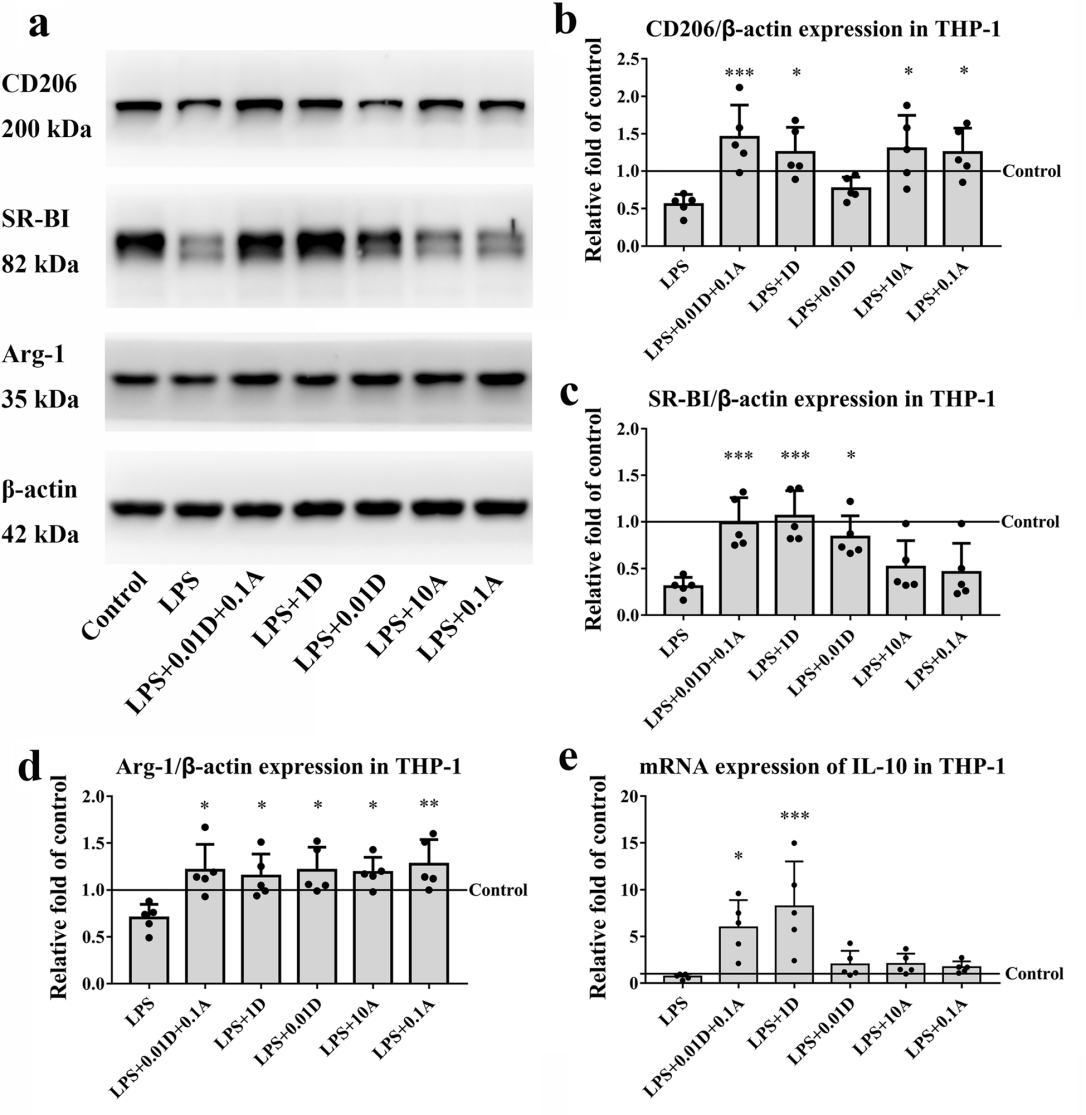
Effects of ATO and DEX on the expression of M2 markers. **a** Representative images of western blots showing CD206, SR-BI and Arg-1 expression. **b** Quantification of CD206, one-way ANOVA, *P* = 0.001; compared with the LPS group, Bonferroni test, ^***^P = 0.003, ^*^P < 0.05. LPS+0.01D+0.1A group vs. LPS+0.01D group, unpaired Student’s *t* test, *P* = 0.009. **c** Quantification of SR-BI, one-way ANOVA, *P* < 0.001; compared with the LPS group, Bonferroni test, ^***^P < 0.005, ^*^P = 0.044. LPS+0.01D+0.1A group vs. LPS+0.1A group, unpaired Student’s *t* test, *P* = 0.021. **d** Quantification of Arg-1, one-way ANOVA, *P* = 0.006; compared with the LPS group, Bonferroni test, ^**^P = 0.007, ^*^P < 0.05. **e** The mRNA expression of IL-10, one-way ANOVA, *P* < 0.001; compared with the LPS group, the Bonferroni test, ^***^P = 0.001, ^*^P = 0.031. LPS+0.01D+0.1A group vs. LPS+0.1A group, unpaired Student’s *t* test, *P* = 0.012, LPS+0.01D+0.1A group vs. LPS+0.01D group, unpaired Student’s *t* test, *P* = 0.026. *Note*: A, atorvastatin; Arg-1, Arginase-1; D, dexamethasone; SR-B1, Scavenger receptor class B type I

The expressions of the M2 markers CD206, the scavenger receptor class B type I (SR-BI), arginase-1 (Arg-1), and IL-10 were significantly increased after treatment with either ATO combined with DEX or 1 μM DEX alone, but only CD206 and Arg-1 increased in macrophages treated with 0.1 μM ATO; meanwhile, SR-BI and Arg-1 increased in those treated with 0.01 μM DEX (Fig. 4).

### DEX enhanced the activity of ATO for inhibiting the release of angiogenic factors from cultured macrophages

**Fig. 5:**
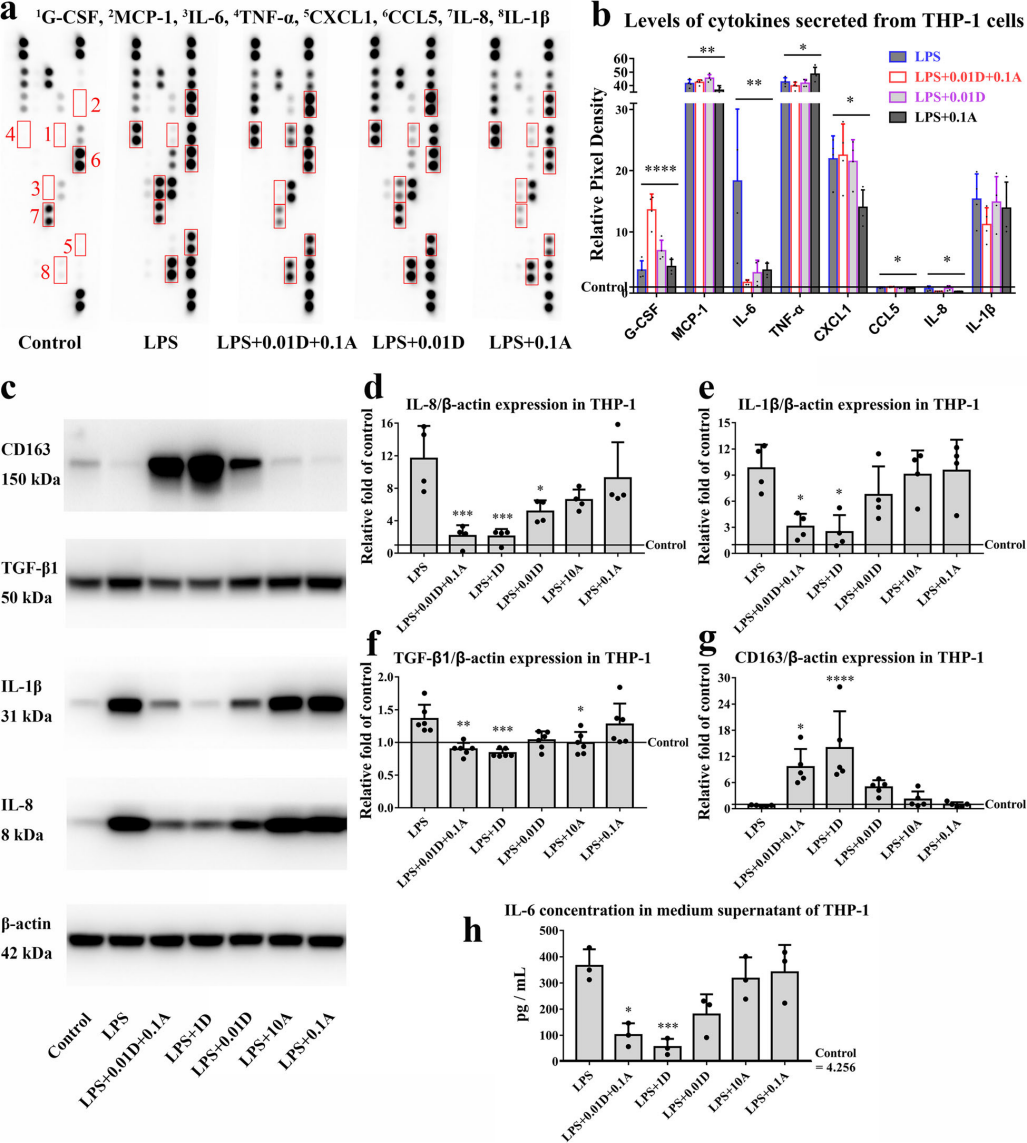
Effects of ATO and DEX on the levels of cytokines and chemokines secreted from macrophages. **a**, **b** The expression profiles of cytokines and chemokines are shown by the cytokine array. One-way ANOVA, ^****^P < 0.001, ^**^P < 0.01, ^*^P < 0.05. **c** Representative images of western blots showing IL-8, IL-1β, TGF-β1 and CD163 expression. **d** Quantification of IL-8, one-way ANOVA, *P* < 0.001; compared with the LPS group, Bonferroni test, ^***^P = 0.001, ^*^P = 0.038. LPS+0.01D+0.1A group vs. LPS+0.1A group, unpaired Student’s *t* test, ^*^P = 0.021, LPS+0.01D+0.1A group vs. LPS+0.01D group, unpaired Student’s *t* test, ^*^P = 0.02. **e** Quantification of IL-1β, one-way ANOVA, *P* = 0.002; compared with the LPS group, Bonferroni test, ^*^P < 0.05. LPS+0.01D+0.1A group vs. LPS+0.1A group, unpaired Student’s *t* test, ^*^P = 0.015. **f** Quantification of TGF-β1, one-way ANOVA, *P* = 0.001; compared with the LPS group, Bonferroni test, ^***^P = 0.003, ^**^P = 0.009, ^*^P = 0.04. LPS+0.01D+0.1A group vs. LPS+0.1A group, unpaired Student’s *t* test, ^*^P = 0.032. **g** Quantification of CD163, one-way ANOVA, *P* < 0.001; compared with the LPS group, Bonferroni test, ^****^P < 0.001, ^*^P = 0.025. LPS+0.01D+0.1A group vs. LPS+0.1A group, unpaired Student’s *t* test, ^**^P = 0.009, LPS+0.01D+0.1A group vs. LPS+0.01D group, unpaired Student’s *t* test, ^*^P = 0.047. **h** ELISA revealing the expression of IL-6, one-way ANOVA, *P* < 0.001; compared with the LPS group, the Bonferroni test, ^***^P = 0.003, ^*^P = 0.01. LPS+0.01D+0.1A group vs. LPS+0.1A group, unpaired Student’s *t* test, ^*^P = 0.021. *Note*: A, atorvastatin; D, dexamethasone

Levels of G-CSF, MCP-1, IL-6, TNF-a, CXCL1, CCL5 and IL-8 secreted by macrophages were notably changed after treatment with ATO and DEX (Fig. 5a, b). The variation trend of these targets, which play an important role in the pathology and cure of CSDH, was further confirmed by immunoblots (Fig. 5c–g) or ELISAs (Fig. 5h). IL-8, IL-1β, TGF-β1 and IL-6 were markedly decreased after treatment with either 1 μM DEX alone or ATO combined with DEX, the latter of which was superior to ATO monotherapy. IL-8 in macrophages treated with 0.01 μM DEX and TGF-β1 in those treated with 10 μM ATO were slightly decreased, as compared to those treated with LPS (Fig. 5d–h). CD163 was increased in macrophages treated with ATO and DEX, consistent with findings from flow cytometry (Fig. 5g).

**Fig. 6:**
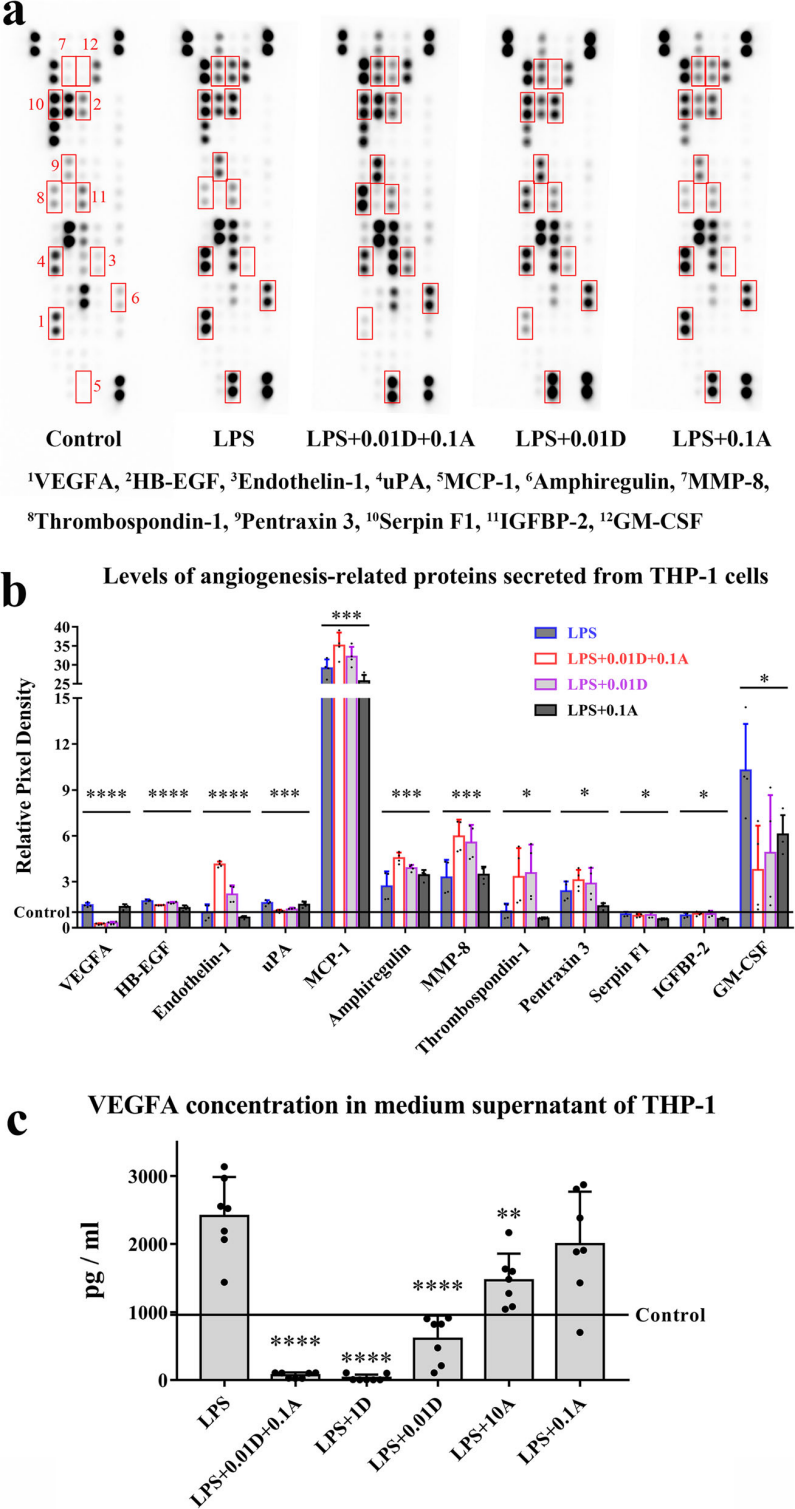
Effects of ATO and DEX on the levels of angiogenesis-related proteins secreted from THP-1 macrophages. **a**, **b** The expression profiles of angiogenesis-related proteins are shown by angiogenesis array, one-way ANOVA, ^****^P < 0.001, ^***^P < 0.005, ^*^P < 0.05. **c** ELISA revealing the expression of VEGFA regulated by ATO and DEX, one-way ANOVA, *P* < 0.001; compared with the LPS group, Bonferroni test, ^****^P < 0.001, ^**^P = 0.005. LPS+0.01D+0.1A group vs. LPS+0.1A group, unpaired Student’s *t* test, ^***^P = 0.001, LPS+0.01D+0.1A group vs. LPS+0.01D group, unpaired Student’s *t* test, ^**^P = 0.006. *Note*: A, atorvastatin; D, dexamethasone

We next investigated the effect of ATO and DEX on the expression of angiogenesis-related proteins. There were notable changes in levels of VEGFA, HB-EGF, ET-1, uPA, MCP-1, amphiregulin, MMP-8, thrombospondin-1, pentraxin 3, serpin F1, IGFBP-2 and GM-CSF secreted by macrophages after the ATO and DEX combined treatment. The angiogenesis inhibitor thrombospondin-1 and the vascular maturation promoting factors ET-1 and pentraxin 3 were significantly elevated by the combined therapy, whereas the expression of VEGFA decreased, as compared to those treated with LPS (Fig. 6a, b). The combined treatment decreased both the basal and LPS-induced VEGFA release in macrophages, and this effect was greater than those with either 0.01 μM DEX or 0.1 μM ATO monotherapy (Fig. 6c).

### Effects of ATO or DEX on the apoptosis of macrophages

**Fig. 7:**
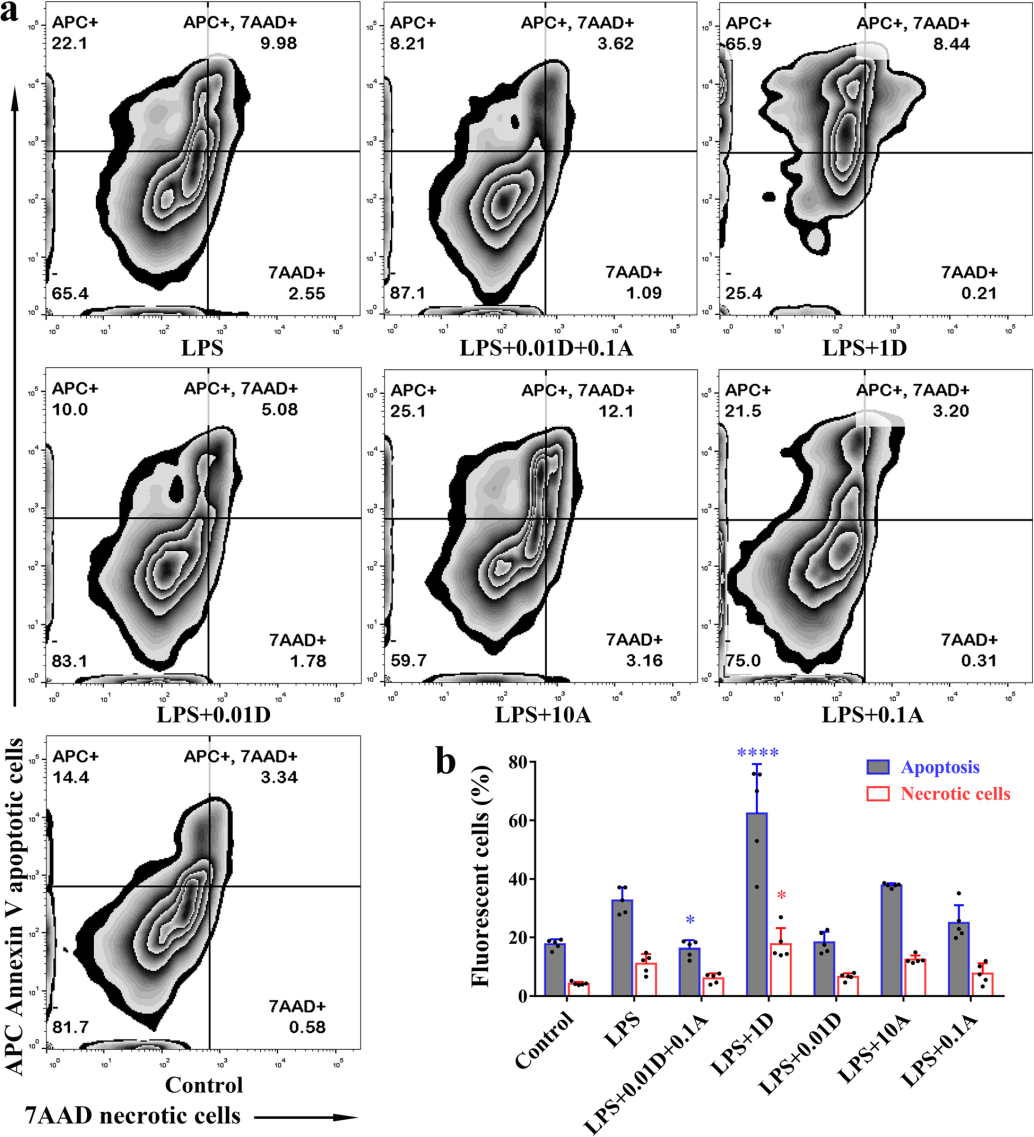
The effect of ATO or DEX on the apoptosis of macrophages. **a** Representative images of macrophage apoptosis. **b** The differences of apoptosis among groups, one-way ANOVA, *P* < 0.001; Bonferroni test, ^****^P < 0.001, ^*^P = 0.039. The differences of necrotic cells among groups, one-way ANOVA, *P* < 0.001; Bonferroni test, ^*^P = 0.042. *Note*: ATO, atorvastatin; DEX, dexamethasone

The percentage of apoptotic cells significantly increased after treatment with 1 μM DEX but decreased when treated with DEX combined with ATO in comparison to those treated with LPS (Fig. 7). Although not statistically significant, apoptosis in the 0.01 μM DEX-treated group or 0.1 μM ATO-treated group also trended lower, whereas the high dose of ATO monotherapy did not cause a significant increase in apoptosis. Similar trends were also found for necrotic cells, but only the 1 μM DEX-treated group reached statistical significance (Fig. 7).

### VEGFA in haematoma fluids and serum samples of CSDH patients

**Fig. 8:**
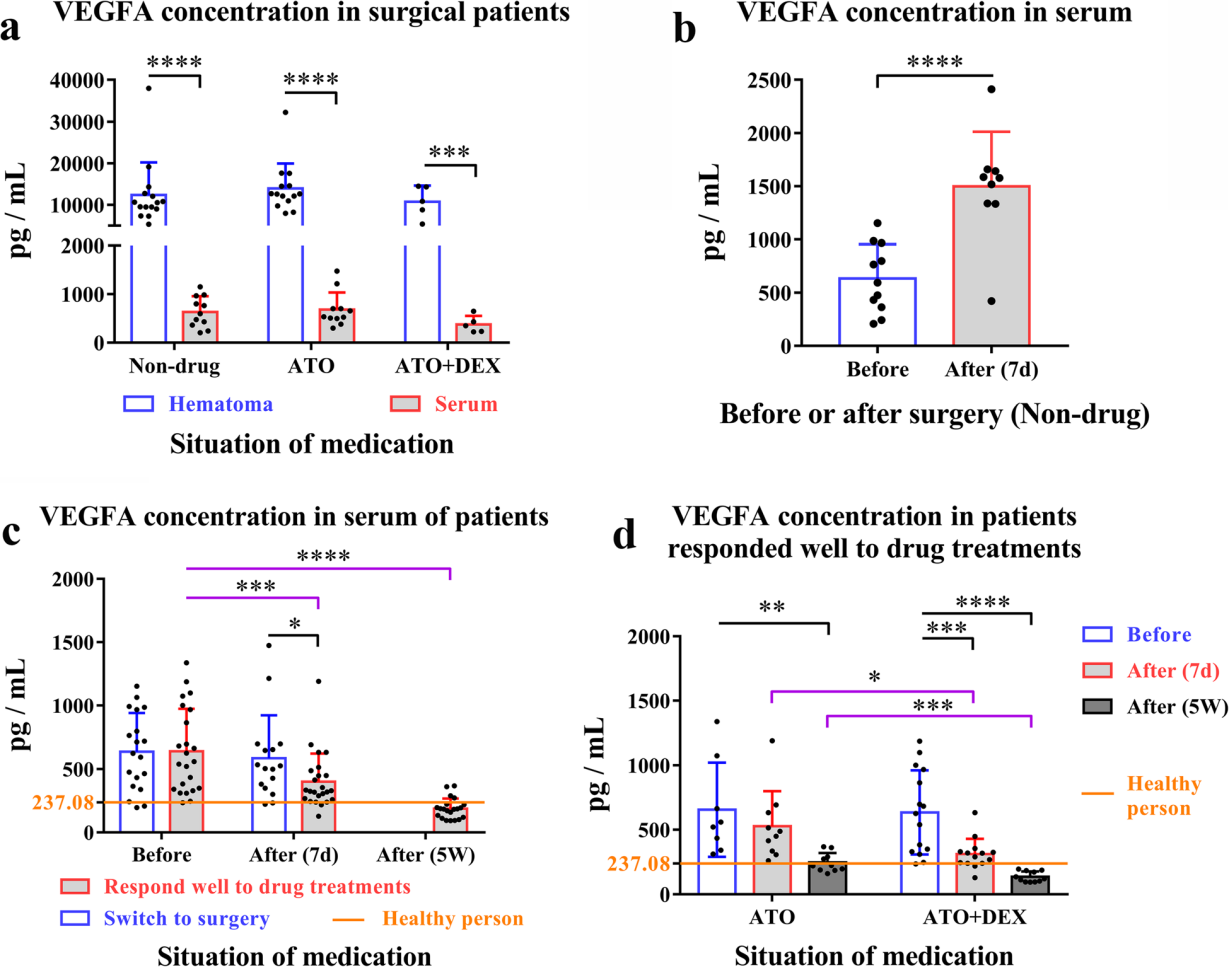
The concentrations of VEGF in the haematoma and serum quantified by ELISA. **a** The concentrations of VEGFA in surgical patients, unpaired Student’s *t* test, ^****^P < 0.001, ^***^P = 0.004. **b** The changes of VEGFA in serum after surgery, unpaired Student’s *t* test, ^****^P < 0.001. **c** The concentrations of VEGFA in conservatively treated patients who have good efficacy or switched to surgery. Conservative treatment and good prognosis, one-way ANOVA, *P* < 0.001; Bonferroni test, before vs. after (5 weeks), ^****^P < 0.001; before vs. after (7 days), ^***^P = 0.003. After (7 days) (switch to surgery vs. conservative treatment and good prognosis, unpaired Student’s *t* test, ^*^P = 0.043). **d** Changes of VEGFA in patients treated with a combination regimen or ATO monotherapy. ATO group, one-way ANOVA, *P* = 0.007; Bonferroni test, before vs. after (5 weeks), ^**^P = 0.007. ATO + DEX group, one-way ANOVA, *P* < 0.001; Bonferroni test, before vs. after (5 weeks), ^****^P < 0.001; before vs. after (7 days), ^***^P = 0.001. After (7 days) (ATO vs. ATO + DEX, unpaired Student’s *t* test, ^*^P = 0.013). After (5 weeks) (ATO vs. ATO + DEX, unpaired student’s *t* test, ^***^P = 0.001). *Note*: ATO, atorvastatin; DEX, dexamethasone; non-drug, patients undergoing surgery directly without conservative treatment

VEGFA, which promotes CSDH [4, 24], showed the most significant decrease after the combined therapy, suggesting that it could be an important biomarker for CSDH. Mean levels of VEGFA in haematoma fluids of CSDH patients undergoing surgery were 12,802.99 pg/ml, which was 21.14 times higher than that of serum samples (605.59 pg/ml, Fig. 8a). Serum VEGFA increased by 136.18% on the seventh day post-surgery (Fig. 8b). Serum VEGFA of patients who responded well to the drug treatment was markedly reduced at 7 days and fifth week during the trial, whereas serum VEGFA was not changed in patients who were switched to surgery (Fig. 8c). For patients who received ATO monotherapy, serum VEGFA levels were reduced after 5 weeks of treatment. Patients with the combined treatment had reduced serum levels of VEGFA at day 7 and week 5, as compared to those receiving ATO monotherapy (Fig. 8d).

ET-1 in haematoma fluid was lower than that in serum samples of patients who received surgery during the trial (Figure S9a). There was no significant increase of ET-1 in haematoma fluid or serum samples of patients after about 7 days of drug treatment (Figure S9b). However, ET-1 secreted by macrophages was significantly increased after treatment with 0.1 μM ATO combined with 0.01 μM DEX or 1 μM DEX alone (Figure S9c).

## Discussion

ATO has been extensively documented to have the anti-inflammatory activity that is independent of its lipid-lowering effects [32, 33] and has also been identified as a substrate for peripheral blood mononuclear cells in vitro [34]. However, several studies have suggested that ATO may not be able to penetrate the blood–brain barrier [35, 36], raising the question about how ATO treatment reduce CSDH. Here, we demonstrated that ATO orally administered to patients with CSDH was detected in the haematoma fluids as well as in cultured macrophages. Haematoma-derived ATO was significantly higher than ATO detected in serum samples from CSDH patients. We also showed that DEX given together with ATO significantly increased the level of ATO in both haematoma fluids and serum samples of CSDH patients, DEX also promoted the intracellular delivery of ATO to cultured macrophages, which are detected in the interstitial tissue of the CSDH membrane, where they mediate inflammation and serve as the main source of VEGFA [24, 26, 37]. We further showed that macrophages were also present in haematoma fluids, with greater numbers of M1 macrophages than M2 macrophages. This macrophage phenotypic profile was regulated by ATO and this regulatory effect was enhanced by DEX, reducing the production and accumulation of VEGFA, which has been associated with recurrent CSDH [4, 25].

Consistent with previous reports [33, 38], we found that ATO achieved its anti-inflammatory and anti-angiogenic activities with a slow kinetic process, potentially taking up to 8 weeks for patients with intense inflammation in haematoma [16]. The prolonged treatment could potentially increase the risk of drug-related complications and noncompliance [20]. By enhancing the effect of ATO, DEX can significantly shorten the treatment regimen in controlling inflammation and preventing aberrant angiogenesis [3]. This notion is supported by the finding that anti-inflammatory and anti-angiogenic therapy can prevent CSDH expansion by blocking the release of VEGFA [26].

To explore the molecular mechanisms underlying these joint effects of ATO and DEX, MS was used to measure drug concentrations in the haematoma, serum and cultured macrophages after drug administration. The present investigation is the first report showing that the concentration of ATO absorbed into the haematoma was significantly higher than that in the serum, which may lay a theoretical basis for the treatment of CSDH with ATO. However, ATO could not be detected in the cerebrospinal fluid after the SDH rat model was treated with ATO (these data will be published in another article), which may be related to the variable results of statins in the treatment of subarachnoid haemorrhage [39]. Moreover, the data suggest a significant increase in ATO concentration following administration together with DEX compared to treatment with ATO alone in the haematoma, serum and cultured macrophages.

We showed in this study that the angiogenesis inhibitor thrombospondin-1 [40] or the vascular maturation factors ET-1 and pentraxin 3 [41, 42] were significantly elevated by combined therapy in this study. These results are consistent with previous reports that ATO inhibits inflammatory angiogenesis and promotes vascular maturation by reducing macrophage infiltration and downregulation of VEGFA, MCP-1, TNF-α or TGF-β1 [43, 44]. Reducing inflammation-induced angiogenesis and increasing the maturation of the neovascularization are associated with haematoma absorption [1, 15]. ATO at high doses (>0.1 μM) but not low doses reduces not only VEGFA plasma levels in coronary artery disease patients but also basal and LPS-induced VEGFA synthesis in human vascular smooth muscle cells and microvascular endothelial cells [45, 46]. However, this ATO effect on VEGFA expression may be cell type specific, as low doses of ATO upregulated VEGFA expression in HUVECs [47]. We did not detect significant changes in all CSDH-related proteins in LPS-activated macrophages with and without treatment with 0.1 μM ATO. However, ATO at 10 μM reduced M1 macrophages and suppressed the expression of TGF-β1 and VEGFA, but the effect was less significant than those treated with both ATO and DEX, suggesting that DEX may have provided additional benefit beyond the increase of ATO absorption.

DEX is suggested to exert its anti-inflammatory effect by regulating the differentiation of macrophages and inhibiting the release of angiogenic cytokines, but its action of DEX in CSDH patients remains poorly understood [4]. High doses of DEX have been reported to resolve CSDH or reduce the risk of recurrence after initial surgery [14]. However, randomized controlled trial confirmed that the notable side effect profiles of DEX (12 mg/day for 3 weeks or 16 mg/day for 2 weeks, followed by tapering) outweigh the benefits leading to serious adverse complications [10, 11, 15]. In this study, we showed that DEX at low doses can promote the effect of ATO without significant toxic effects, suggesting the synergy between the two drugs.

Our study also suggests that VEGFA could serve as a biomarker for evaluating CSDH development, progression and response to treatment [48]. This finding is consistent with extensive previous reports that a high level of VEGFA in haematoma fluid and that outer membrane promotes CSDH growth [24] and associated with a higher rate of recurrence [25]. It has long been suggested that the bur hole irrigation surgery interrupts the self-perpetuating vicious cycle by removing fibrinolytic enhancers and restoring the normal haemostatic balance [49]. For example, the surgical drainage of haematoma could reduce VEGFA accumulated in haematoma fluids [9, 26]. However, we found that serum VEGFA was significantly increased after surgery, which may be due to the release of VEGFA in haematoma fluid caused by the destruction of the blood–brain barrier or other complex mechanisms after surgery. Regardless of its cause, the high VEGFA level in blood samples may be a potential risk factor for CSDH recurrence after surgery, because of VEGFA promoting CSDH formation. In this regard, ATO combined DEX given post-surgery could potential reduce the risk for CSDH recurrence, as previous studies suggested [14, 50].

Different signalling pathways have been suggested to play roles in the formation and progression of CSDH [4]. In addition, angiogenic factors, such as IL-1β, IL-6, IL-8 and TNF-α [22], have been found to have significantly elevated levels in haematoma fluids, as compared with peripheral blood samples of patients with CSDH [23, 51] and are correlated with an increased risk of CSDH recurrence [25, 52]. ATO and DEX block these pro-CSDH factors through synergistic actions.

There are limitations in the study. The role of VEGFA in the treatment and prognosis of CSDH needs to be further confirmed by animal experiments and randomized clinical trials. The specific signalling pathways involved in inhibiting the release of VEGFA or other related angiogenic factors by the combination therapy need to be further clarified. One potential pitfall of the study is that macrophages and other blood cells are accumulated over a prolonged period of time in haematoma fluids. During their accumulation, fresh cells enter into the haematoma fluid whereas existing cells undergo apoptosis and are removed from the fluid. As such, our data present a snap shot of cellular profile at the time when haematoma fluid was collected.

## Conclusions

We have provided evidence that orally taken ATO entered into haematoma fluids of patients with CSDH to suppress macrophage-medicated proinflammatory and proangiogenic activates. DEX enhances the action of ATO in reducing CSDH by promoting its entrance to haematoma fluids and macrophages. The combined therapy alters the macrophage phenotype, promoting the transition from the proinflammatory phenotype to the anti-inflammatory phenotype, and regulating the level of VEGFA and other inflammatory angiogenic factors.

## Supplementary Information

**Additional file 1: File S1.** The inclusion and exclusion criteria. **Table S1.** RT-PCR Primers used in this study. **Table S2.** Baseline characteristics and outcome of CSDH patients treated with a combination regimen or ATO monotherapy. **Table S3.** Baseline characteristics and outcomes of conservatively treated patients who have good efficacy or switched to surgery. **Table S4.** The functions of these proteins identified, but not specifically discussed in the manuscript. **Figure S1.** o-ATO and p-ATO in CSDH patients. **Figure S2.** Concentrations of ATO and DEX in HUVEC. **Figure S3.** Effects of ATO and DEX on expression of drug transport and catabolism-related proteins in macrophages. **Figure S4.** Monocytes and macrophages in the haematoma of CSDH patients. **Figure S5.** The differentiation of THP-1 cells into macrophages stimulated by PMA. **Figure S6.** LPS can effectively simulate the effect of haematoma on THP-1 macrophages. **Figure S7.** Regulation of ATO and DEX on the morphological changes of THP-1 macrophages. **Figure S8.** The effect of ATO and DEX on the MFI of CD86 and CD163 in macrophages. **Figure S9.** The concentrations of ET-1 in the haematoma, serum and medium supernatant quantified by ELISA.

## Data Availability

All data generated and analyzed during this study are included in this published article and its supplementary files.

## Abbreviations

ATO: Atorvastatin
CSDH: Chronic subdural haematoma
CT: Computed tomography
CYP3A4: Cytochrome P450 3A4
DEX: Dexamethasone
ET-1: Endothelin-1
LPS: Lipopolysaccharide
MRI: Magnetic resonance imaging
MS: Mass spectrometry
o-ATO: Ortho-hydroxy-atorvastatin
OATP1A2: Organic anion-transporting polypeptide 1A2
PMA: Phorbol-12-myristate-13-acetate
p-ATO: Para-hydroxy-atorvastatin
UPLC: Ultra-high-performance liquid chromatography
THP-1: Human acute monocytic leukaemia cell line
VEGFA: Vascular endothelial growth factor
