# Supplementary material for: Dexamethasone enhances the efficacy of atorvastatin in inhibiting excessively inflammation-induced abnormal angiogenesis by regulating macrophages
Source: J Neuroinflammation. 2021 Sep 15;18:203. doi: 10.1186/s12974-021-02257-1 (PMC8444603; doi:10.1186/s12974-021-02257-1)
Supplement: Supplementary file 1 — Additional file 1: File S1. The inclusion and exclusion criteria. Table S1. RT-PCR Primers used in this study. Table S2. Baseline characteristics and outcome of CSDH patients treated with a combination regimen or ATO monotherapy. Table S3. Baseline characteristics and outcomes of conservatively treated patients who have good efficacy or switched to surgery. Table S4. The functions of these proteins identified, but not specifically discussed in the manuscript. Figure S1. o-ATO and p-ATO in CSDH patients. Figure S2. Concentrations of ATO and DEX in HUVEC. Figure S3. Effects of ATO and DEX on expression of drug transport and catabolism-related proteins in macrophages. Figure S4. Monocytes and macrophages in the haematoma of CSDH patients. Figure S5. The differentiation of THP-1 cells into macrophages stimulated by PMA. Figure S6. LPS can effectively simulate the effect of haematoma on THP-1 macrophages. Figure S7. Regulation of ATO and DEX on the morphological changes of THP-1 macrophages. Figure S8. The effect of ATO and DEX on the MFI of CD86 and CD163 in macrophages. Figure S9. The concentrations of ET-1 in the haematoma, serum and medium supernatant quantified by ELISA. [file 12974_2021_2257_MOESM1_ESM.zip › Revised additional files-JNEU-D-21-00254.docx]

**Additional files (File S1, Tables S1-4, Fig. S1-9)**

**File S1** The inclusion and exclusion criteria.

The inclusion criteria were adult (age ≥ 18 years) men and nonpregnant women; diagnosed as primary supratentorial CSDH with minimal midline shift (< 1 cm) by Computed Tomography (CT) or Magnetic Resonance Imaging (MRI); the mild or moderate patients who are not at risk of cerebral hernia and not in need of immediate operation; Markwalder grading scale–Glasgow Coma Scale (MGS-GCS) < 3 and no previous surgery for CSDH; and patients who refused surgery. Patients were excluded if they were allergic to statins; had a secondary cause of the haematoma (e.g., tumor or bleeding diathesis), severe comorbidity or liver disease; had received a statin or steroid within the last week; had diabetes mellitus with poorly controlled blood glucose; had uncontrolled hypertension or infection; had already participated in clinical trials in the previous 4 weeks; or had historical and current bleeding or thrombosis with or without treatment (or were taking prophylactic antiplatelet medications).

**Table S1** RT-PCR Primers used in this study.

| Gene | Primer name (sequence) |
| --- | --- |
| IL-10 | F(5ˊ-GTTGTTAAAGGAGTCCTTGCTG-3ˊ) |
|  | R(5ˊ-TTCACAGGGAAGAAATCGATGA-3ˊ) |
| P-glycoprotein | F(5ˊ-GCCAAAGCCAAAATATCAGC-3ˊ) |
|  | R(5ˊ-TTCCAATGTGTTCGGCAT-3ˊ) |
| CYP3A4 | F(5ˊ-GGCTTCATCCAATGGACTGCATAAAT-3ˊ) |
|  | R(5ˊ-TCCCAAGTATAACACTCTACACAGACAA-3ˊ) |
| GAPDH | F(5ˊ-GGCCTCCAAGGAGTAAGACC-3ˊ) |
|  | R(5ˊ-AGGGGAGATTCAGTGTGGTG-3ˊ) |

NOTE. CYP3A4, Cytochrome P450 3A4.

**Table S2** Baseline characteristics and outcome of CSDH patients treated with a combination regimen or ATO monotherapy.

As it’s very difficult to collect serum samples from the mild patients who took oral medication at home, we only successfully obtained the samples from 5 participants of these patients. For the hospitalized patients whom we got the consent to collect samples, 24 patients failed in conservative treatment and switched to surgery, and 61 conservatively treated patients achieved good efficacy. Among these conservatively treated patients who have good efficacy, the samples from 31 patients were successfully collected at the point of therapeutic time required for this study. For the patients failed in conservative treatment and switched to surgery, almost no serum samples were obtained at the fifth week of medication before surgery, because the median duration of medication before surgery was 8.5 days. Among the total of 60 patients whose clinical samples were successfully collected, 28 patients received atorvastatin monotherapy at the outset, and the remaining 32 patients came from the combination therapy group.

| **CSDH patients** | **ATO monotherapy (n=28)** | **Combination regimen (n=32)** | ***P*** |
| --- | --- | --- | --- |
| Gender: male | 22 (78.57%) | 26 (81.25%) | 0.796^a^ |
| Age (years) | 69.00 (65.25, 81.00) | 71.00 (65.50, 83.75) | 0.528^b^ |
| CSDH with TBI history | 12 (42.86%) | 11 (34.38%) | 0.500^a^ |
| Past medical history |  |  |  |
| Hypertension | 13 (46.43%) | 13 (40.63%) | 0.651^a^ |
| Diabetes | 6 (21.43%) | 10 (31.25%) | 0.391^a^ |
| Hyperlipidaemia | 2 (7.14%) | 5 (15.63%) | 0.307^a^ |
| Baseline on admission |  |  |  |
| Haematoma volume (mL) | 75.00 (41.34, 93.43) | 74.79 (51.12, 121.26) | 0.281^b^ |
| GCS score | 15.00 (15.00, 15.00) | 15.00 (15.00, 15.00) | 0.256^b^ |
| MGS-GCS score | 1.00 (1.00, 1.00) | 1.00 (1.00, 1.00) | 0.185^b^ |
| Outcome (Switched to surgery) | 15 (53.57%) | 9 (28.13%) | **0.045^a^*** |
| Duration of medication before surgery | 8.00 (5.00, 14.00) | 9.00 (4.50, 25.00) | 0.770^b^ |

NOTE. CSDH, chronic subdural haematoma; MGS-GCS, Markwalder grading scale–Glasgow Coma Scale; Switched to surgery: patients failed in conservative treatment and switched to surgery; TBI, traumatic brain injury; ^a^Pearson chi-square test; ^b^Mann–Whitney U test; *Bold denotes statistical significance (*P* < 0.05)

**Table S3** Baseline characteristics and outcomes of conservatively treated patients who have good efficacy or switched to surgery.

| **CSDH patients** | **Switch to Surgery (n=24)** | **Conservative treatment and good prognosis (n=36)** | ***P*** |
| --- | --- | --- | --- |
| Gender: male | 23 (95.83%) | 25 (69.44%) | **0.012^a^*** |
| Age (years) | 76.00 (68.25, 81.00) | 68.50 (61.75, 82.50) | 0.141^b^ |
| CSDH with TBI history | 8 (33.33%) | 15 (41.67%) | 0.515^a^ |
| Past medical history |  |  |  |
| Hypertension | 12 (50.00%) | 14 (38.89%) | 0.395^a^ |
| Diabetes | 7 (29.17%) | 9 (25.00%) | 0.721^a^ |
| Hyperlipidaemia | 3 (12.50%) | 4 (11.11%) | 0.870^a^ |
| Baseline on admission |  |  |  |
| Heamatoma volume (mL) | 83.93 (75.00, 99.19) | 59.00 (40.73, 110.82) | 0.050^b^ |
| GCS score | 15.00 (15.00, 15.00) | 15.00 (15.00, 15.00) | 0.129^b^ |
| MGS-GCS score | 1.00 (1.00, 1.00) | 1.00 (1.00, 1.00) | 0.238^b^ |
| Outcomes |  |  |  |
| Haematoma volume (mL) | 100.00 (85.50, 130.00) | 32.45 (21.05, 49.50) | **<0.001^b^*** |
| GCS score | 15.00 (13.25, 15.00) | 15.00 (15.00, 15.00) | **0.001^b^*** |
| MGS-GCS score | 1.00 (1.00, 2.00) | 0.00 (0.00, 0.00) | **<0.001^b^*** |

NOTE. CSDH, chronic subdural haematoma; MGS-GCS, Markwalder grading scale–Glasgow Coma Scale; Switched to surgery: patients failed in conservative treatment and switched to surgery; TBI, traumatic brain injury; ^a^Pearson chi-square test; ^b^Mann–Whitney U test; *Bold denotes statistical significance (*P* < 0.05)

**Table S4** The functions of these proteins identified, but not specifically discussed in the manuscript.

| Name | Functions |
| --- | --- |
| G-CSF | Human Granulocyte-Colony-Stimulating Factor (G-CSF) is 20 kD glycoprotein containing internal disulfide bonds. It induces the survival, proliferation, and differentiation of neutrophilic granulocyte precursor cells and it functionally activates mature blood neutrophils. Among the family of colony-stimulating factors, G-CSF is the most potent inducer of terminal differentiation to granulocytes and macrophages of leukemic myeloid cell lines. The synthesis of G-CSF can be induced by bacterial endotoxins, TNF, Interleukin-1, and GM-CSF. Prostaglandin E2 inhibits the synthesis of G-CSF. In epithelial, endothelial, and fibroblastic cells secretion of GCSF is induced by Interleukin-17. |
|  |  |
| MCP-1 | MCP-1/JE/ CCL2, is a chemokine that binds the receptor CCR2 and induces the chemoattraction of mononuclear cells. It induces the activation of monocytes, NK cells, lymphocytes, and basophils. Additionally, CCL2 promotes Th2 polarization in CD4+ T cells, and CCL2-mediated recruitment of monocytes to sites of inflammation contributes to disease severity in atherosclerosis, multiple sclerosis, and allergic asthma. |
|  |  |
| TNF-α | TNF-alpha(Tumor necrosis factor alpha) plays a central role in inflammation, immune system development,apoptosis, and lipid metabolism. TNF-alpha was first identified as a cytotoxic factor produced by macrophages capable of killing mouse tumor cells. |
| CXCL1 | CXCL1, also known as KC and GRO alpha, is a CXC family chemokine that is expressed in fibroblasts, macrophages, and endothelial cells and interacts with CXCR2. CXCL1 is a potent neutrophil attractant and activator. It also promotes angiogenesis by inducing VEGF production by neutrophils. |
|  |  |
| CCL5 | CCL5(Regulated upon Activation, Normal T cell Expressed and presumably Secreted), also known as RANTES, is a member of the "CC" subfamily of chemokines. It plays a primary role in the inflammatory immune response via its ability to chemoattract leukocytes and modulate their function. The cDNA for RANTES was initially discovered by subtractive hybridization as a T cell specific sequence. |
|  |  |
| HB-EGF | The transmembrane form of HB-EGF is a juxtacrine growth and adhesion factor and is uniquely the receptor for diphtheria toxin. HB-EGF gene expression is highly regulated, for example by cytokines, growth factors, and transcription factors such as MyoD. HB-EGF has been implicated as a participant in a variety of normal physiological processes such as blastocyst implantation and wound healing, and in pathological processes such as tumor growth, SMC hyperplasia and atherosclerosis. |
|  |  |
| uPA | u-Plasminogen Activator (uPA) is a serine protease that converts plasminogen to plasmin, with roles in a variety of normal and pathological processes that include cell migration and tissue destruction. uPA is a potent marker of invasion and metastasis in a variety of human cancers including breast, stomach, colon, bladder, ovarian, brain, and endometrium. |
|  |  |
| Amphiregulin | Amphiregulin (AR) is an EGF family growth factor that is released as a soluble protein following proteolytic cleavage of its transmembrane precursor. Amphiregulin is expressed by numerous carcinoma cell lines and epithelial cells of the colon, stomach, breast, ovary and kidney. It acts through ErbB family of receptor to stimulate the proliferation of keratinocytes, mammary epithelial cells, fibroblasts, astrocytes and glial cells. Amphiregulin can also inhibit the growth of certain tumor cells. |
| MMP-8 | The matrix metalloproteinases (MMPs) consist of 24 known human zinc proteases with essential roles in breaking down components of the extracellular matrix (ECM). |
|  |  |
| Serpin F1 | The human serpin superfamily consists of at least 35 members that target not only serine proteases, but also selected cysteine proteases and non-protease proteins. Serpins bind the protease active site resulting in a major conformational rearrangement that traps the enzyme in a covalent acyl-enzyme intermediate. As protease inhibitors, serpins have an array of functions including regulating blood clotting, the complement pathway, extracellular matrix remodeling, and cell motility. |
| IGFBP-2 | Insulin-like Growth Factor Binding Protein 2 (IGFBP2) is a member of the family of high-affinity binding proteins (IGFBP1-6) and appears to play a governing role in Insulin-like Growth Factor (IGF) regulation in the central nervous system[1]. Insulin-like Growth Factor-Binding Proteins (IGFBPs) modulate the actions of secreted insulin-like growth facts (IGFs) by binding to them and increase the IGF halflife in the extracellular milieu and circulation by sequestering them in this bound form. |
|  |  |
| GM-CSF | GM‑CSF was initially characterized as a factor that can support the in vitro colony formation of granulocyte‑macrophage progenitors. It is also a growth factor for erythroid, megakaryocyte, and eosinophil progenitors. GM‑CSF is produced by a number of different cell types (including T cells, B cells, macrophages, mast cells, endothelial cells, fibroblasts, and adipocytes) in response to cytokine or inflammatory stimuli. On mature hematopoietic cells, GM‑CSF is a survival factor for and activates the effector functions of granulocytes, monocytes/macrophages, and eosinophils. |


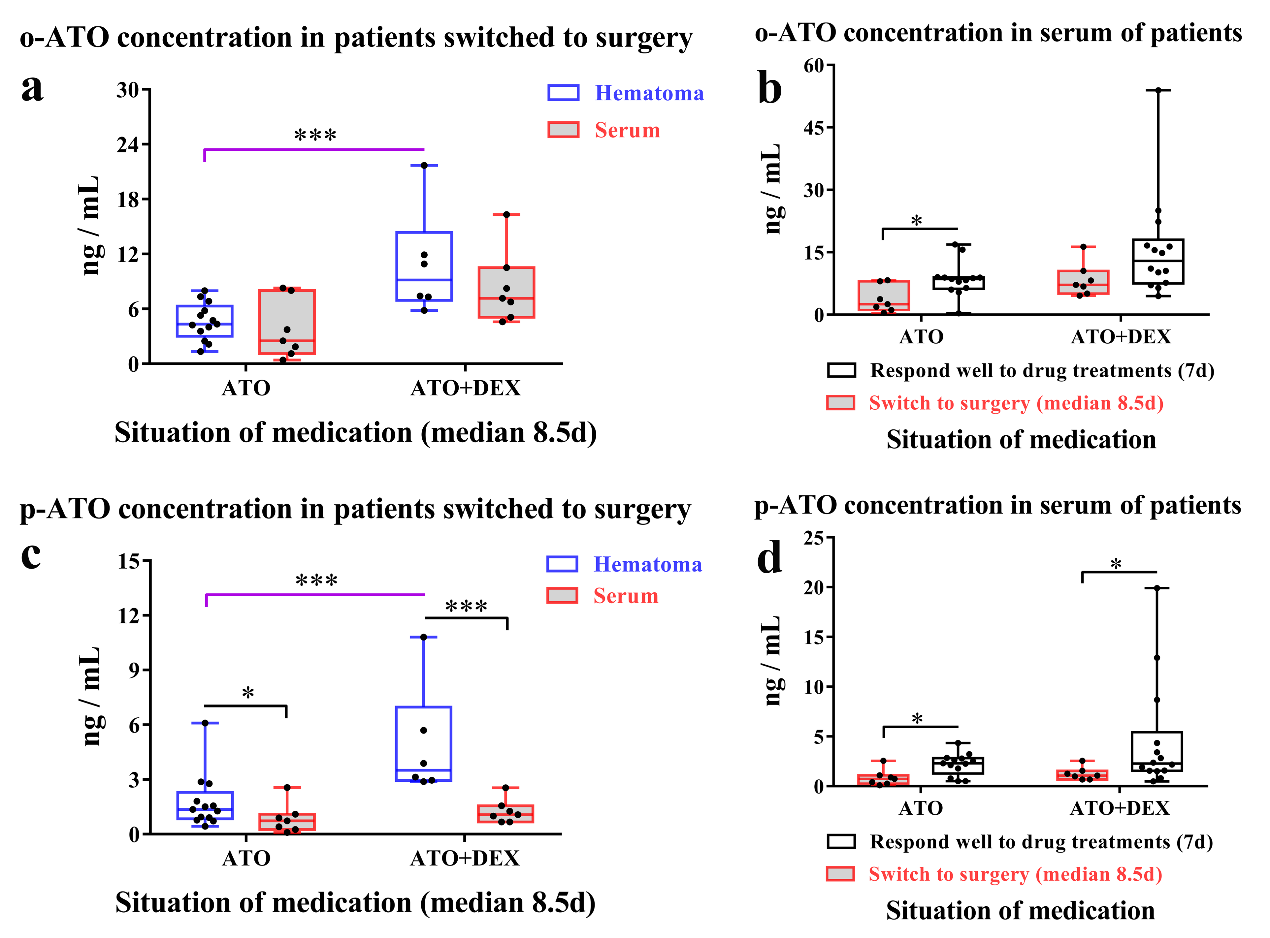


**Fig. S1 o-ATO and p-ATO in CSDH patients.** (**a**) o-ATO concentration in haematoma and serum of patients switched to surgery, Haematoma (ATO vs. ATO + DEX, Mann–Whitney U test, ^***^*P* = 0.002); Serum (ATO vs. ATO + DEX, Mann–Whitney U test, *P* = 0.053). (**b**) Serum o-ATO in patients who responded well to the drug treatments and those who switched to surgery, ATO (Switch to surgery vs. Respond well to drug treatments, Mann–Whitney U test, ^*^*P* = 0.019); Switch to surgery (ATO vs. ATO + DEX, Mann–Whitney U test, *P* = 0.053); Respond well to drug treatments (ATO vs. ATO + DEX, Mann–Whitney U test, *P* = 0.054). (**c**) p-ATO concentration in haematoma and serum of patients switched to surgery, ATO (Haematoma vs. Serum, Mann–Whitney U test, ^*^*P*=0.046); ATO + DEX (Haematoma vs. Serum, Mann–Whitney U test, ^***^*P*=0.001); Haematoma (ATO vs. ATO + DEX, Mann–Whitney U test, ^***^*P* = 0.001). (**d**) Serum p-ATO in patients who responded well to the drug treatments and those who switched to surgery, ATO (Switch to surgery vs. Respond well to drug treatments, Mann–Whitney U test, ^*^*P* = 0.019); ATO + DEX (Switch to surgery vs. Respond well to drug treatments, Mann–Whitney U test, ^*^*P* = 0.025). NOTE. ATO, atorvastatin; DEX, dexamethasone; o-ATO, ortho-hydroxy-atorvastatin; p-ATO, para-hydroxy-atorvastatin.


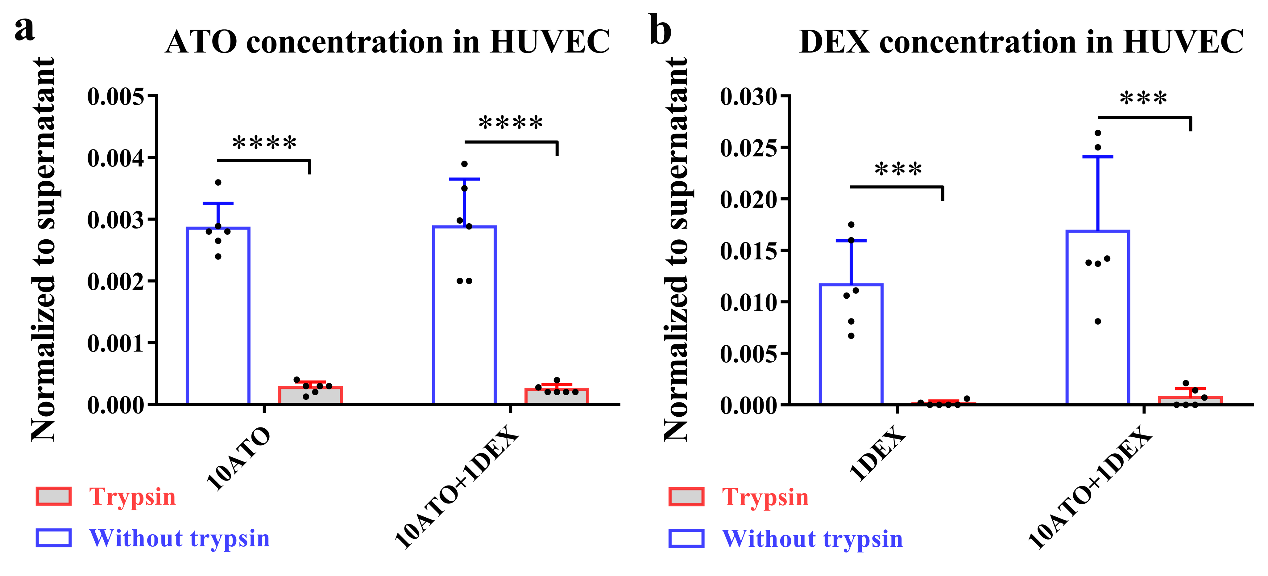


**Fig. S2** **Concentrations of ATO and DEX in HUVEC.** (**a**) The concentration of ATO in HUVEC, 10ATO vs. 10ATO (Trypsin), paired Student’s t-test, ^****^*P* < 0.001; 10ATO + 1DEX vs. 10ATO + 1DEX (Trypsin), paired Student’s t-test, ^****^*P* < 0.001. (**b**) The concentration of DEX in HUVEC, 1DEX vs. 1DEX (Trypsin), paired Student’s t-test, ^***^*P* = 0.001; 10ATO + 1DEX vs. 10ATO + 1DEX (Trypsin), paired Student’s t-test, ^***^*P* = 0.003. NOTE: 10ATO, 10 μM atorvastatin; 10ATO+1DEX, 10 μM atorvastatin and 1 μM dexamethasone; 1DEX, 1 μM dexamethasone; HUVEC, Human umbilical vein endothelial cells.


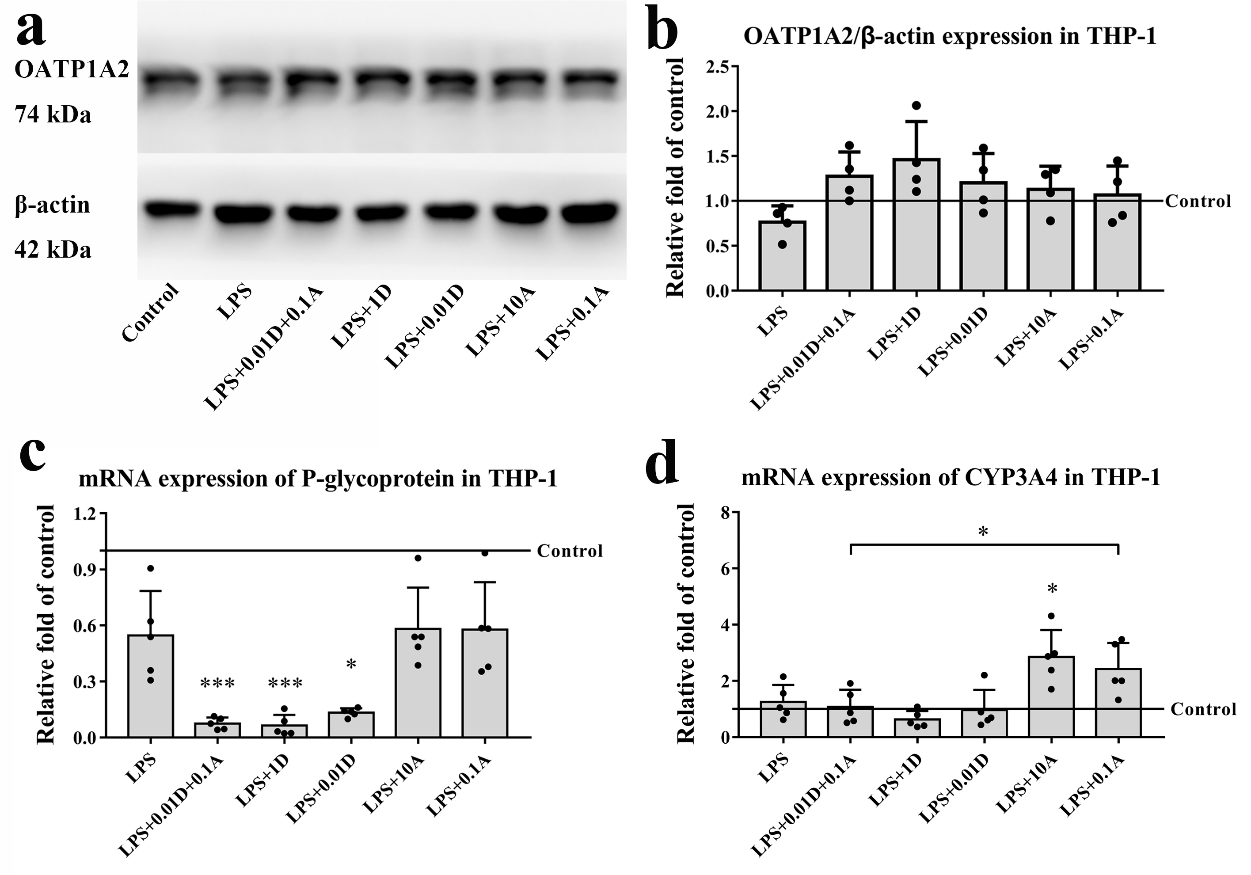


**Fig. S3** **Effects of ATO and DEX on expression of drug transport and catabolism-related proteins in macrophages.** (**a**) Western blot analysis showing the expression of OATP1A2. (**b**) OATP1A2, one-way ANOVA, *P* = 0.089. (**c**) The mRNA expression of P-glycoprotein, one-way ANOVA, *P* < 0.001; compared with the LPS group, the Bonferroni test, ^***^*P* < 0.005, ^*^*P* = 0.012. LPS+0.01D+0.1A group vs. LPS+0.1A group, unpaired Student’s t-test, *P* = 0.002, LPS+0.01D+0.1A group vs. LPS+0.01D group, unpaired Student’s t-test, *P* = 0.01. (**d**) The mRNA expression of CYP3A4, one-way ANOVA, *P* < 0.001; compared with the LPS group, the Bonferroni test, ^*^*P* = 0.027. LPS+0.01D+0.1A group vs. LPS+0.1A group, unpaired Student’s t-test, ^*^*P* = 0.026. NOTE. CYP3A4, Cytochrome P450 3A4; OATP1A2, Organic anion transporting polypeptide 1A2.


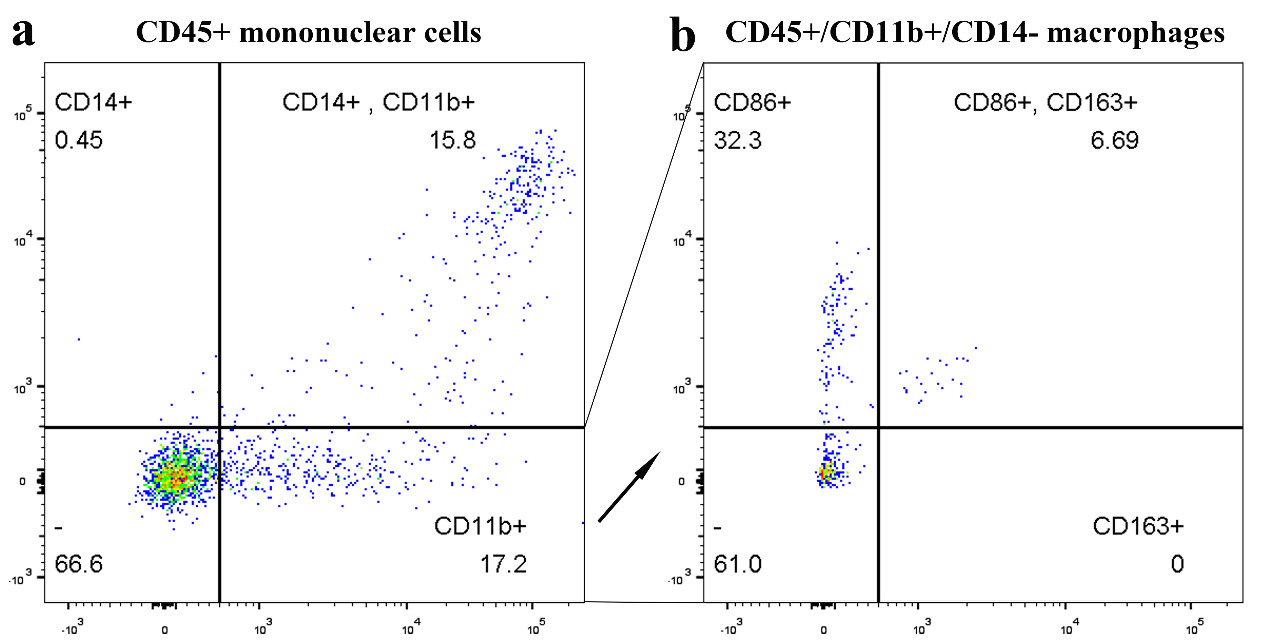


**Fig. S4 Monocytes and macrophages in the haematoma of CSDH patients.** (**a**) Representative image of monocytes and macrophages in mononuclear cells. The CD45+/CD11b+/CD14+ monocytes in haematoma of CSDH patients accounted for an average of 16.13% of CD45+ mononuclear cells. And the CD45+/CD11b+/CD14- macrophages accounted for about 17.6% of CD45+ mononuclear cells. (**b**) Representative image of the differentiation of macrophages. On average, 36.47% M1-polarized macrophages and 8.66% M2-polarized macrophages were indicated in CD45+/CD11b+/CD14- macrophages. NOTE. CSDH, chronic subdural haematoma; PerCP/Cyanine5.5 anti-human CD45 Antibody (304028, BioLegend, USA, 1: 1000); APC anti-human CD14 Antibody (367118, BioLegend, USA, 1: 1000).


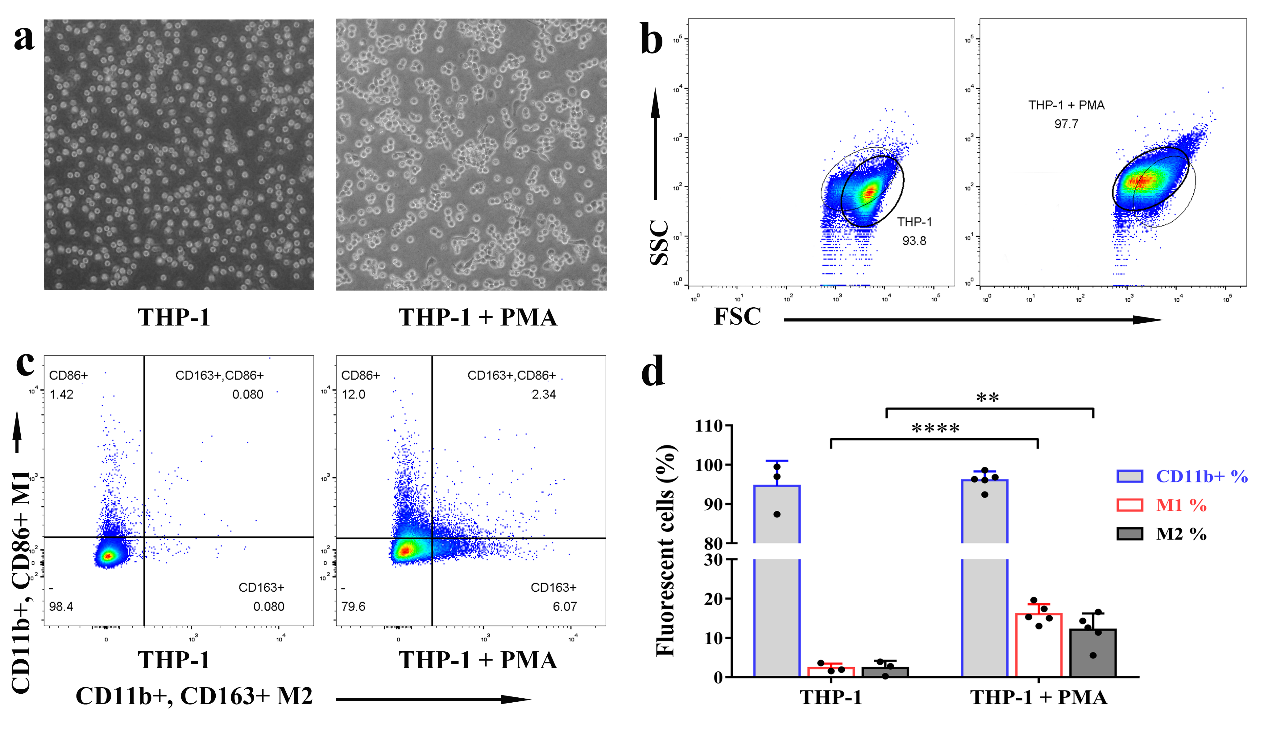


**Fig. S5 The differentiation of THP-1 cells into macrophages stimulated by PMA.** (**a**) Representative images of the morphological changes. (b, c) Representative images of the flow cytometry analysis. (d) Quantification of the THP-1 differentiation into macrophages by flow cytometry. M1 % (THP-1 vs. THP-1 + PMA), Unpaired Student’s t-test, ^****^*P* < 0.001; M2 % (THP-1 vs. THP-1 + PMA), Unpaired Student’s t-test, ^**^*P* = 0.009.


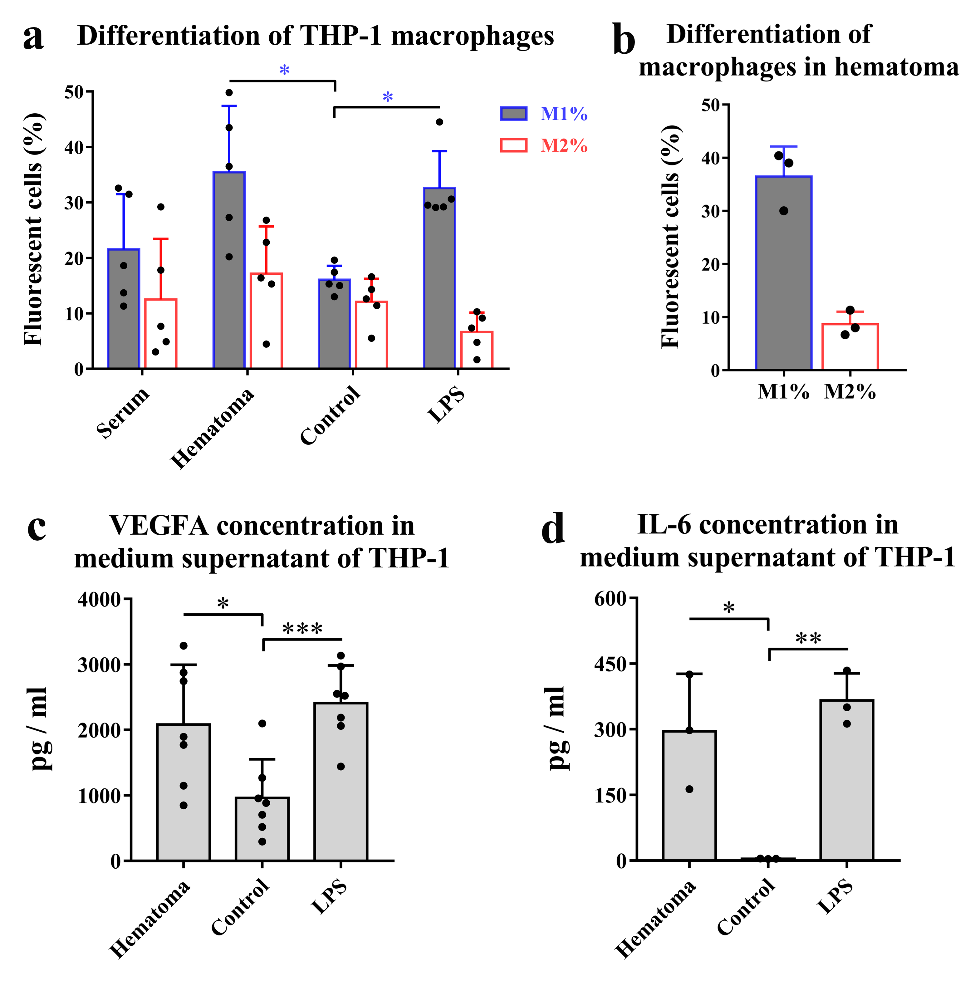


**Fig. S6 LPS can effectively simulate the effect of haematoma on THP-1 macrophages.** (**a**) Flow cytometry analysis for the differentiation of THP-1 macrophages, M1%, One-Way ANOVA analysis, *P* = 0.007; Compared with control group, Bonferroni test, ^*^*P* < 0.05. M2%, One-Way ANOVA analysis, *P* = 0.213. (**b**) Flow cytometry analysis for the differentiation of macrophages in haematoma of CSDH patients. (**c**) ELISA test revealing the expression of VEGFA regulated by haematoma, One-Way ANOVA analysis, *P* = 0.001. Compared with control group, Bonferroni test, ^*^*P* = 0.017, ^***^*P* = 0.001. (**d**) ELISA test revealing the expression of IL-6 regulated by haematoma, One-Way ANOVA analysis, *P* = 0.004. Compared with control group, Bonferroni test, ^*^*P* = 0.016, ^**^*P* = 0.006.


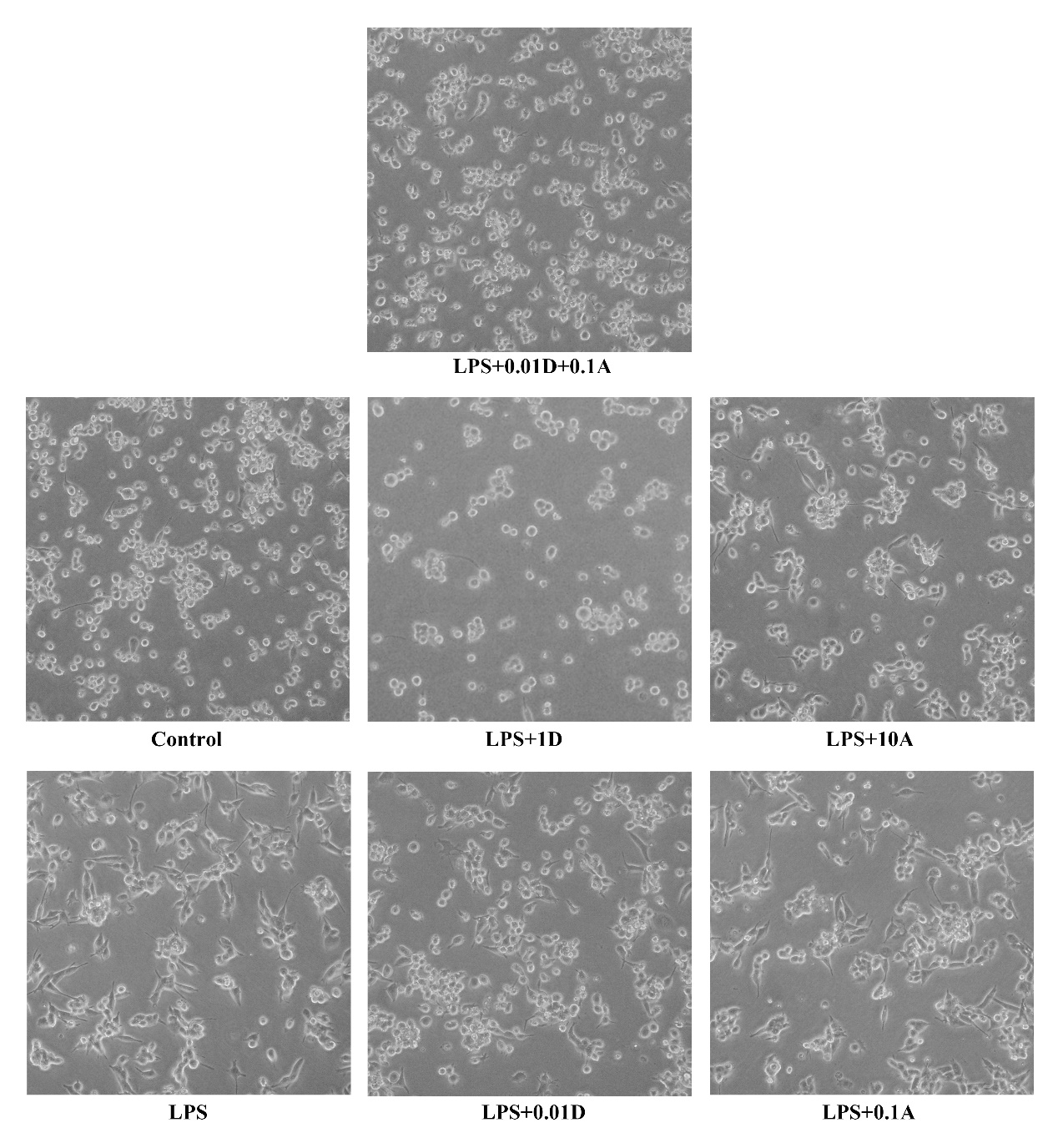


**Fig. S7 Regulation of** **ATO and DEX** **on the morphological changes of** **THP-1 macrophages.** NOTE. A, atorvastatin; D, dexamethasone**.**


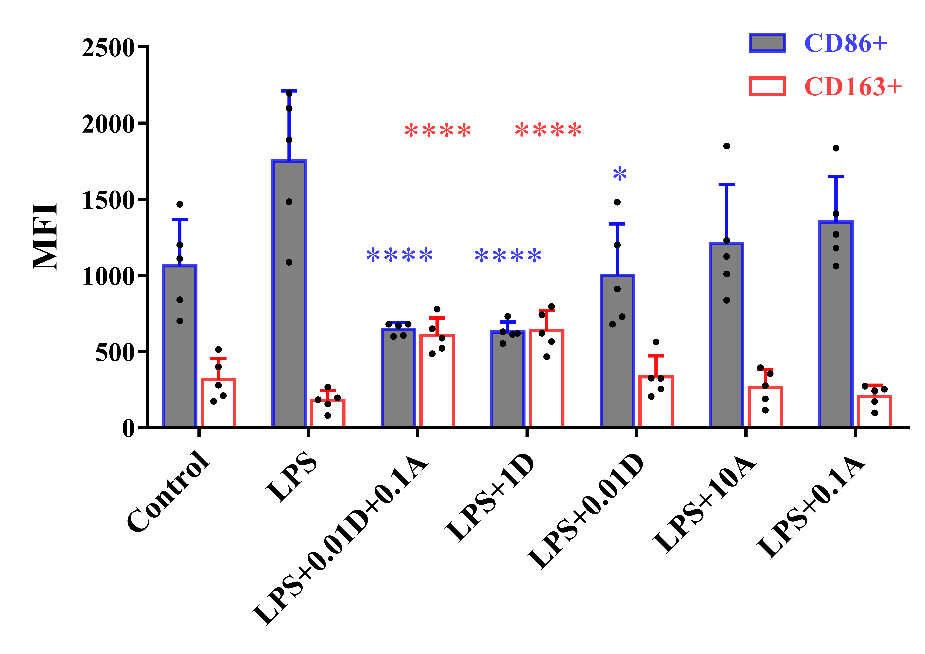


**Fig. S8** **The effect of ATO and DEX on the MFI of CD86 and CD163 in macrophages.** MFI of CD86+ macrophages, one-way ANOVA, *P* < 0.001; compared with the LPS group, Bonferroni test, ^****^*P* < 0.001, ^*^*P* = 0.012. MFI of CD163+ macrophages, one-way ANOVA, *P* < 0.001; compared with the LPS group, Bonferroni test, ^****^*P* < 0.001. NOTE: A, atorvastatin; D, dexamethasone; MFI, Median fluorescence intensity.


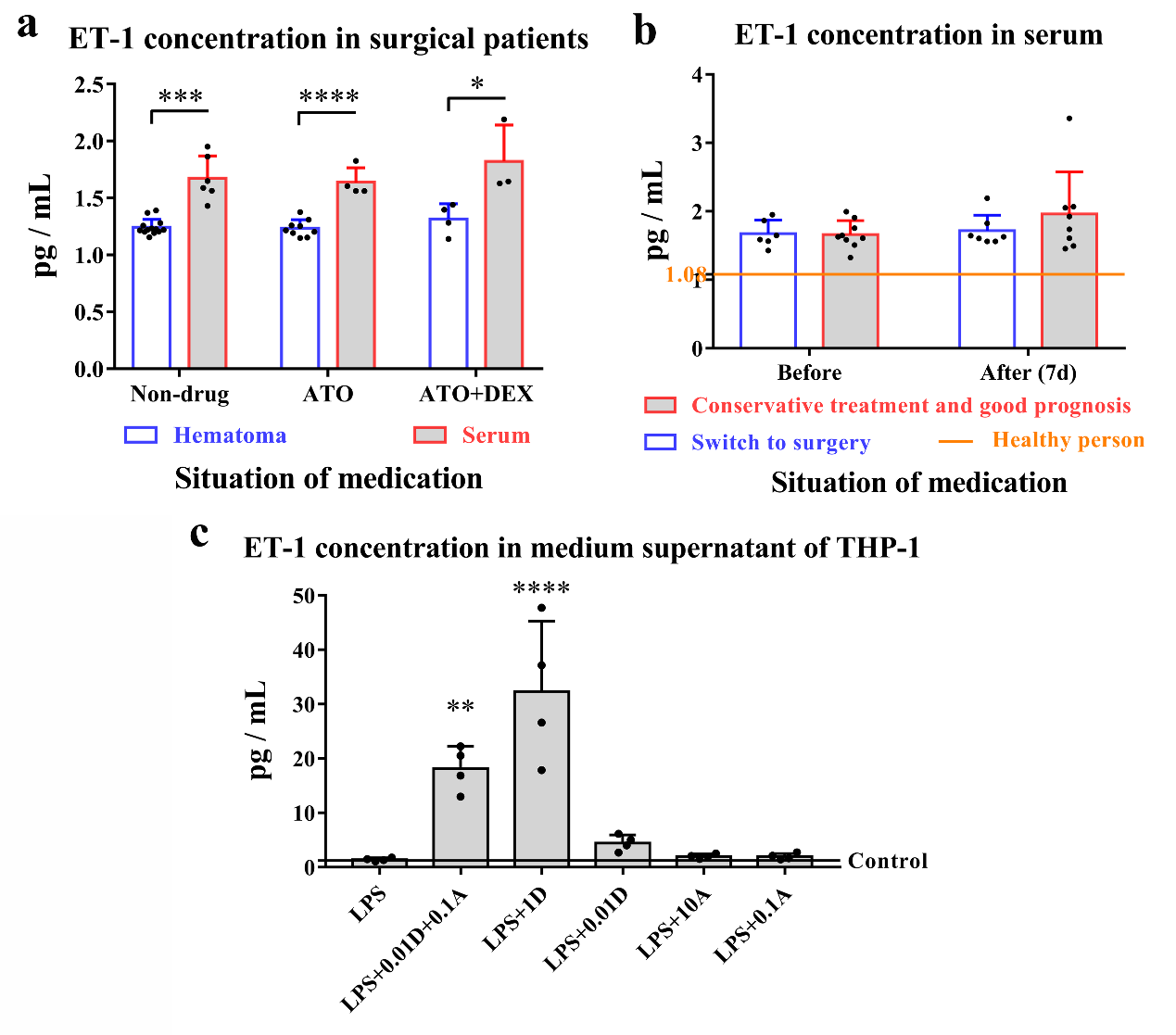


**Fig. S****9** **The concentrations of ET-1 in the** **haematoma, serum and medium supernatant quantified by ELISA.** (**a**) The concentrations of ET-1 in surgical patients, unpaired Student’s t-test, ^****^*P* < 0.001, ^***^*P* = 0.002, ^*^*P* = 0.033. (**b**) The concentrations of ET-1 in conservatively treated patients who have good efficacy or switched to surgery. (**c**) The expression of ET-1 regulated by ATO and DEX, one-way ANOVA, *P* < 0.001; compared with the LPS group, Bonferroni test, ^****^*P* < 0.001, ^**^*P* = 0.007. LPS+0.01D+0.1A group vs. LPS+0.1A group, unpaired Student’s t-test, *P* = 0.004, LPS+0.01D+0.1A group vs. LPS+0.01D group, unpaired Student’s t-test, *P* = 0.001. NOTE: A, atorvastatin; D, dexamethasone; ET-1, endothelin-1; Non-drug, Patients undergoing surgery directly without conservative treatment.
