# Supplementary figures and images for: Dexamethasone enhances the efficacy of atorvastatin in inhibiting excessively inflammation-induced abnormal angiogenesis by regulating macrophages
Source: J Neuroinflammation. 2021 Sep 15;18:203. doi: 10.1186/s12974-021-02257-1 (PMC8444603; doi:10.1186/s12974-021-02257-1)

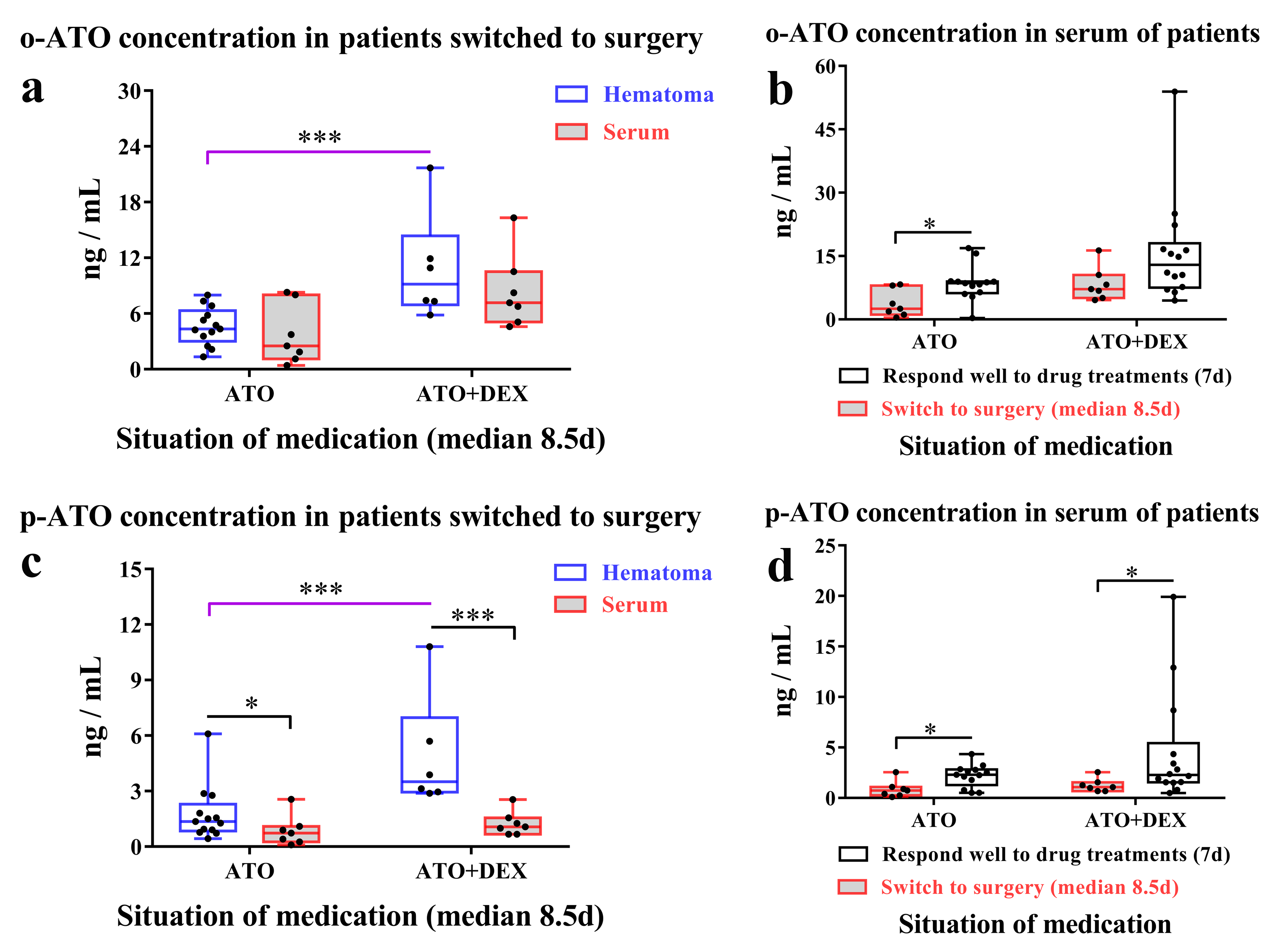

Supplement: Supplementary file 1 — Additional file 1: File S1. The inclusion and exclusion criteria. Table S1. RT-PCR Primers used in this study. Table S2. Baseline characteristics and outcome of CSDH patients treated with a combination regimen or ATO monotherapy. Table S3. Baseline characteristics and outcomes of conservatively treated patients who have good efficacy or switched to surgery. Table S4. The functions of these proteins identified, but not specifically discussed in the manuscript. Figure S1. o-ATO and p-ATO in CSDH patients. Figure S2. Concentrations of ATO and DEX in HUVEC. Figure S3. Effects of ATO and DEX on expression of drug transport and catabolism-related proteins in macrophages. Figure S4. Monocytes and macrophages in the haematoma of CSDH patients. Figure S5. The differentiation of THP-1 cells into macrophages stimulated by PMA. Figure S6. LPS can effectively simulate the effect of haematoma on THP-1 macrophages. Figure S7. Regulation of ATO and DEX on the morphological changes of THP-1 macrophages. Figure S8. The effect of ATO and DEX on the MFI of CD86 and CD163 in macrophages. Figure S9. The concentrations of ET-1 in the haematoma, serum and medium supernatant quantified by ELISA. [file 12974_2021_2257_MOESM1_ESM.zip › Fig. S1(updated).tif]

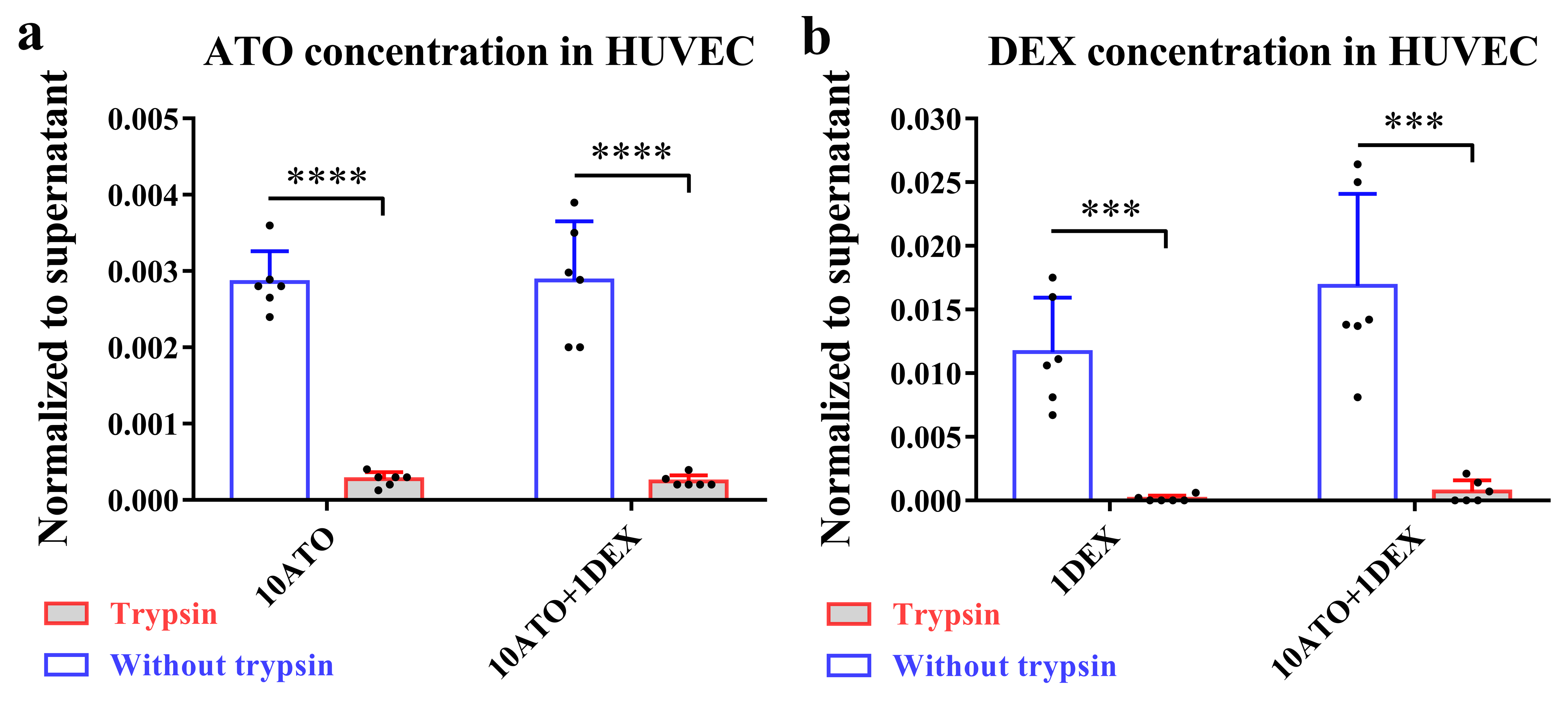

Supplement: Supplementary file 1 — Additional file 1: File S1. The inclusion and exclusion criteria. Table S1. RT-PCR Primers used in this study. Table S2. Baseline characteristics and outcome of CSDH patients treated with a combination regimen or ATO monotherapy. Table S3. Baseline characteristics and outcomes of conservatively treated patients who have good efficacy or switched to surgery. Table S4. The functions of these proteins identified, but not specifically discussed in the manuscript. Figure S1. o-ATO and p-ATO in CSDH patients. Figure S2. Concentrations of ATO and DEX in HUVEC. Figure S3. Effects of ATO and DEX on expression of drug transport and catabolism-related proteins in macrophages. Figure S4. Monocytes and macrophages in the haematoma of CSDH patients. Figure S5. The differentiation of THP-1 cells into macrophages stimulated by PMA. Figure S6. LPS can effectively simulate the effect of haematoma on THP-1 macrophages. Figure S7. Regulation of ATO and DEX on the morphological changes of THP-1 macrophages. Figure S8. The effect of ATO and DEX on the MFI of CD86 and CD163 in macrophages. Figure S9. The concentrations of ET-1 in the haematoma, serum and medium supernatant quantified by ELISA. [file 12974_2021_2257_MOESM1_ESM.zip › Fig. S2(updated).tif]

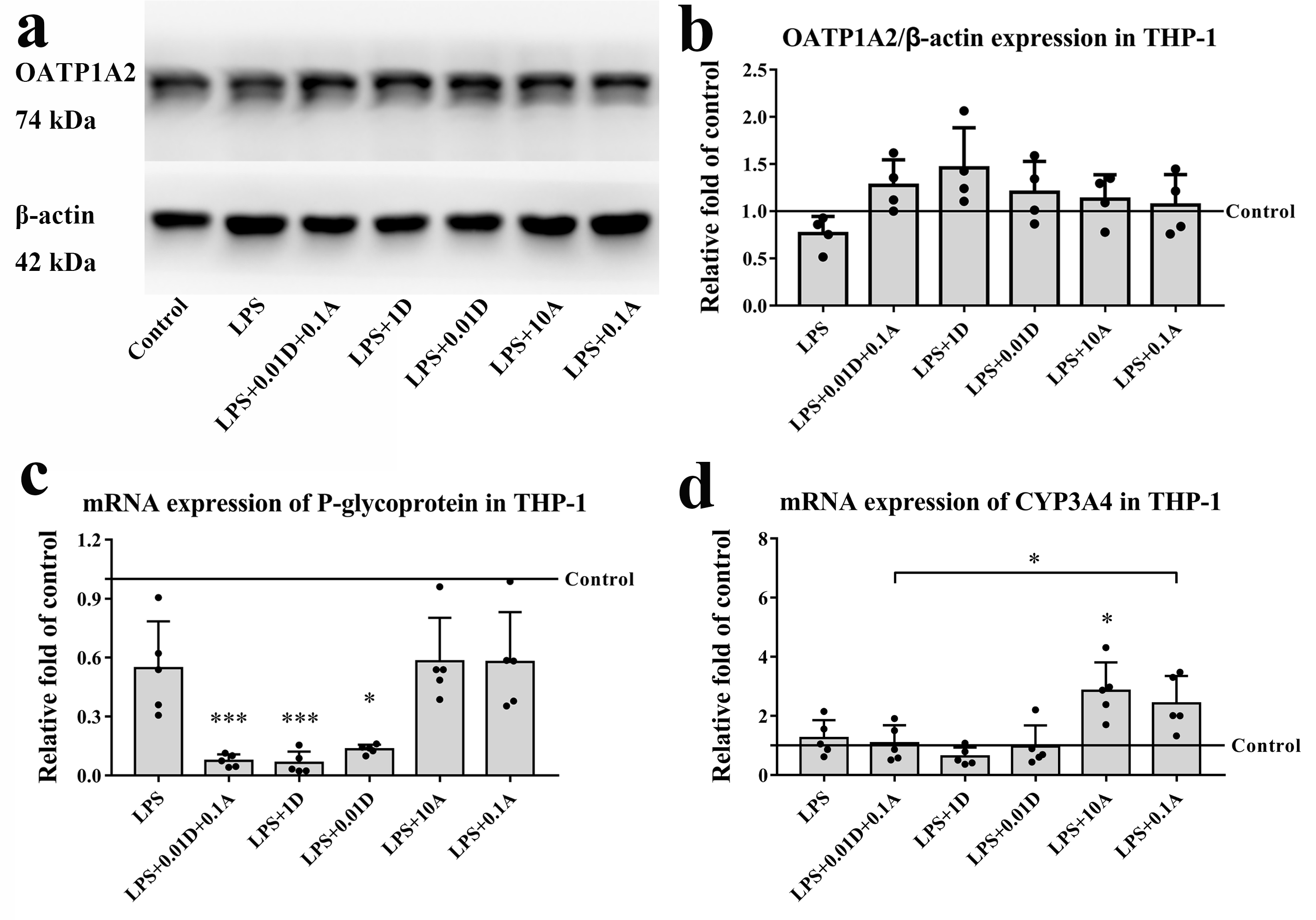

Supplement: Supplementary file 1 — Additional file 1: File S1. The inclusion and exclusion criteria. Table S1. RT-PCR Primers used in this study. Table S2. Baseline characteristics and outcome of CSDH patients treated with a combination regimen or ATO monotherapy. Table S3. Baseline characteristics and outcomes of conservatively treated patients who have good efficacy or switched to surgery. Table S4. The functions of these proteins identified, but not specifically discussed in the manuscript. Figure S1. o-ATO and p-ATO in CSDH patients. Figure S2. Concentrations of ATO and DEX in HUVEC. Figure S3. Effects of ATO and DEX on expression of drug transport and catabolism-related proteins in macrophages. Figure S4. Monocytes and macrophages in the haematoma of CSDH patients. Figure S5. The differentiation of THP-1 cells into macrophages stimulated by PMA. Figure S6. LPS can effectively simulate the effect of haematoma on THP-1 macrophages. Figure S7. Regulation of ATO and DEX on the morphological changes of THP-1 macrophages. Figure S8. The effect of ATO and DEX on the MFI of CD86 and CD163 in macrophages. Figure S9. The concentrations of ET-1 in the haematoma, serum and medium supernatant quantified by ELISA. [file 12974_2021_2257_MOESM1_ESM.zip › Fig. S3(updated).tif]

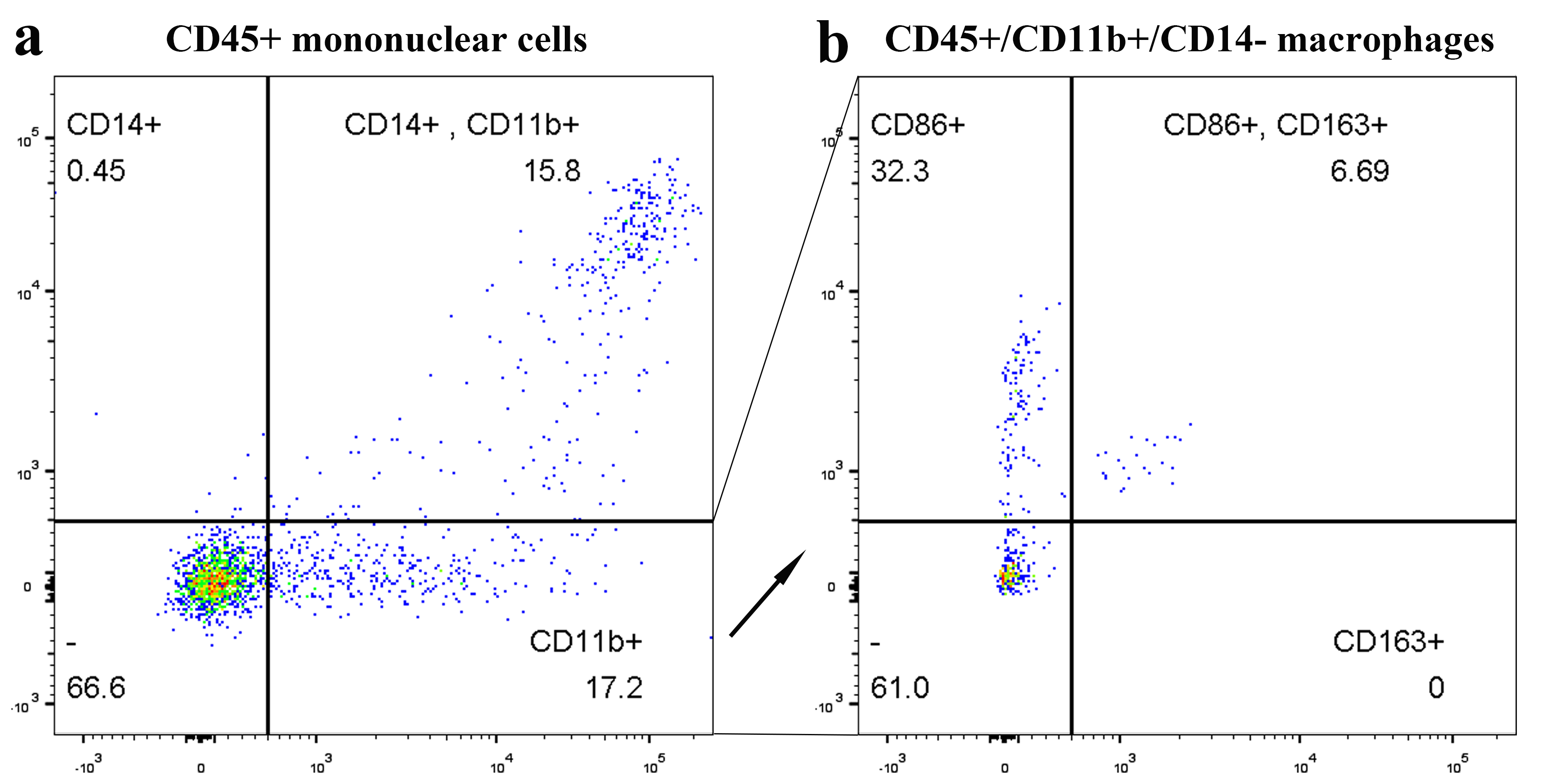

Supplement: Supplementary file 1 — Additional file 1: File S1. The inclusion and exclusion criteria. Table S1. RT-PCR Primers used in this study. Table S2. Baseline characteristics and outcome of CSDH patients treated with a combination regimen or ATO monotherapy. Table S3. Baseline characteristics and outcomes of conservatively treated patients who have good efficacy or switched to surgery. Table S4. The functions of these proteins identified, but not specifically discussed in the manuscript. Figure S1. o-ATO and p-ATO in CSDH patients. Figure S2. Concentrations of ATO and DEX in HUVEC. Figure S3. Effects of ATO and DEX on expression of drug transport and catabolism-related proteins in macrophages. Figure S4. Monocytes and macrophages in the haematoma of CSDH patients. Figure S5. The differentiation of THP-1 cells into macrophages stimulated by PMA. Figure S6. LPS can effectively simulate the effect of haematoma on THP-1 macrophages. Figure S7. Regulation of ATO and DEX on the morphological changes of THP-1 macrophages. Figure S8. The effect of ATO and DEX on the MFI of CD86 and CD163 in macrophages. Figure S9. The concentrations of ET-1 in the haematoma, serum and medium supernatant quantified by ELISA. [file 12974_2021_2257_MOESM1_ESM.zip › Fig. S4(non-updated).tif]

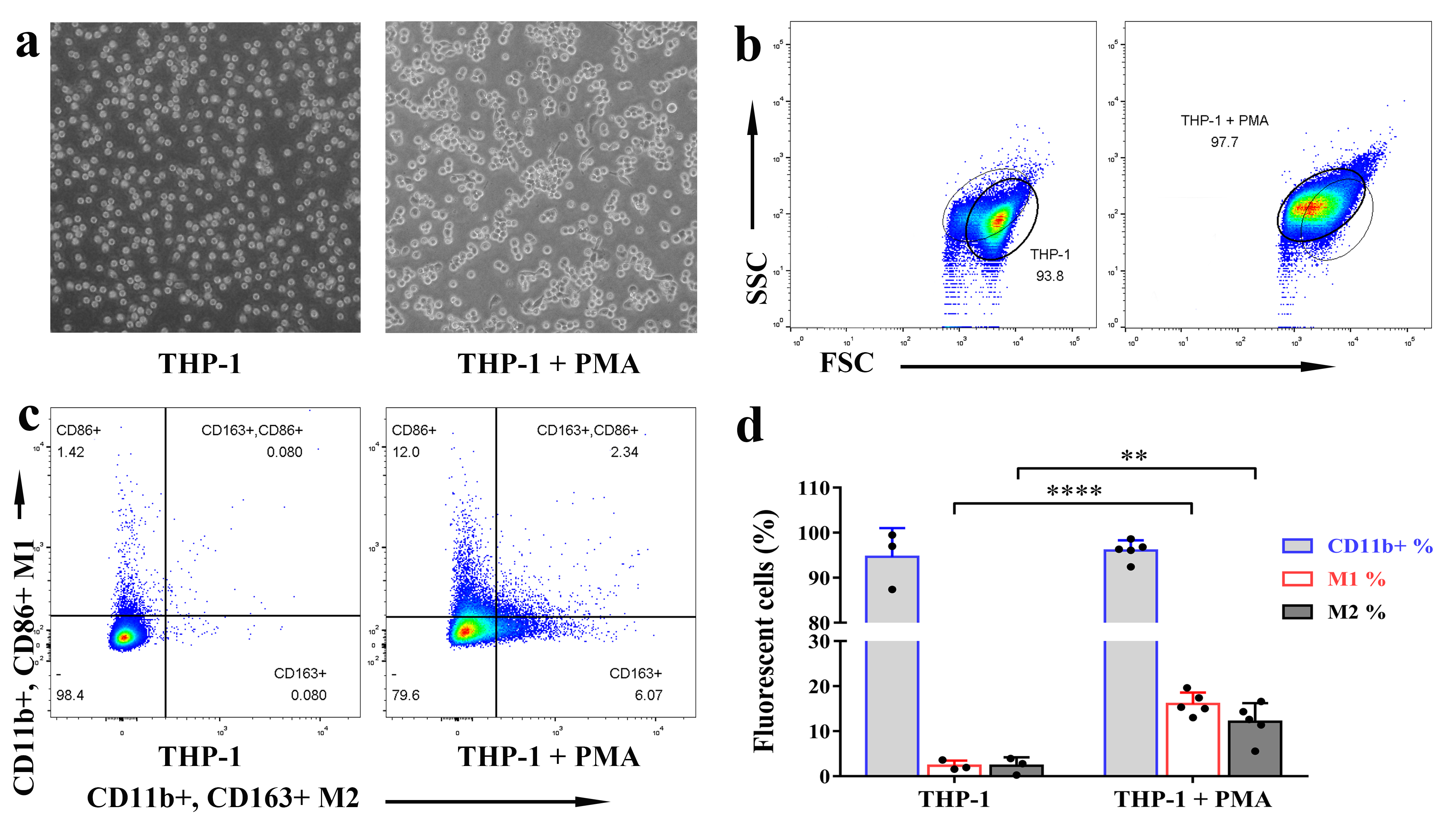

Supplement: Supplementary file 1 — Additional file 1: File S1. The inclusion and exclusion criteria. Table S1. RT-PCR Primers used in this study. Table S2. Baseline characteristics and outcome of CSDH patients treated with a combination regimen or ATO monotherapy. Table S3. Baseline characteristics and outcomes of conservatively treated patients who have good efficacy or switched to surgery. Table S4. The functions of these proteins identified, but not specifically discussed in the manuscript. Figure S1. o-ATO and p-ATO in CSDH patients. Figure S2. Concentrations of ATO and DEX in HUVEC. Figure S3. Effects of ATO and DEX on expression of drug transport and catabolism-related proteins in macrophages. Figure S4. Monocytes and macrophages in the haematoma of CSDH patients. Figure S5. The differentiation of THP-1 cells into macrophages stimulated by PMA. Figure S6. LPS can effectively simulate the effect of haematoma on THP-1 macrophages. Figure S7. Regulation of ATO and DEX on the morphological changes of THP-1 macrophages. Figure S8. The effect of ATO and DEX on the MFI of CD86 and CD163 in macrophages. Figure S9. The concentrations of ET-1 in the haematoma, serum and medium supernatant quantified by ELISA. [file 12974_2021_2257_MOESM1_ESM.zip › Fig. S5(updated).tif]

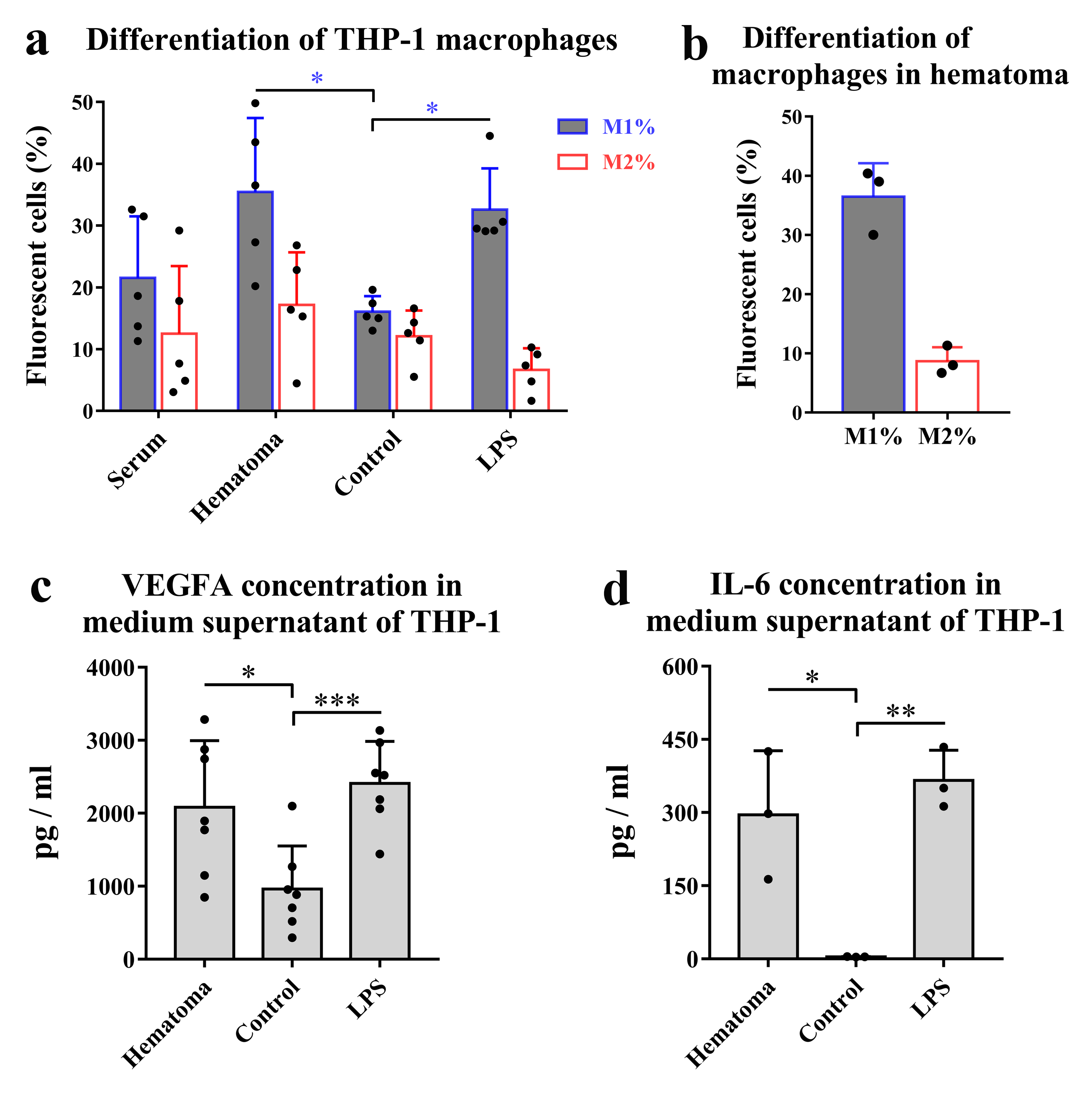

Supplement: Supplementary file 1 — Additional file 1: File S1. The inclusion and exclusion criteria. Table S1. RT-PCR Primers used in this study. Table S2. Baseline characteristics and outcome of CSDH patients treated with a combination regimen or ATO monotherapy. Table S3. Baseline characteristics and outcomes of conservatively treated patients who have good efficacy or switched to surgery. Table S4. The functions of these proteins identified, but not specifically discussed in the manuscript. Figure S1. o-ATO and p-ATO in CSDH patients. Figure S2. Concentrations of ATO and DEX in HUVEC. Figure S3. Effects of ATO and DEX on expression of drug transport and catabolism-related proteins in macrophages. Figure S4. Monocytes and macrophages in the haematoma of CSDH patients. Figure S5. The differentiation of THP-1 cells into macrophages stimulated by PMA. Figure S6. LPS can effectively simulate the effect of haematoma on THP-1 macrophages. Figure S7. Regulation of ATO and DEX on the morphological changes of THP-1 macrophages. Figure S8. The effect of ATO and DEX on the MFI of CD86 and CD163 in macrophages. Figure S9. The concentrations of ET-1 in the haematoma, serum and medium supernatant quantified by ELISA. [file 12974_2021_2257_MOESM1_ESM.zip › Fig. S6(non-updated).tif]

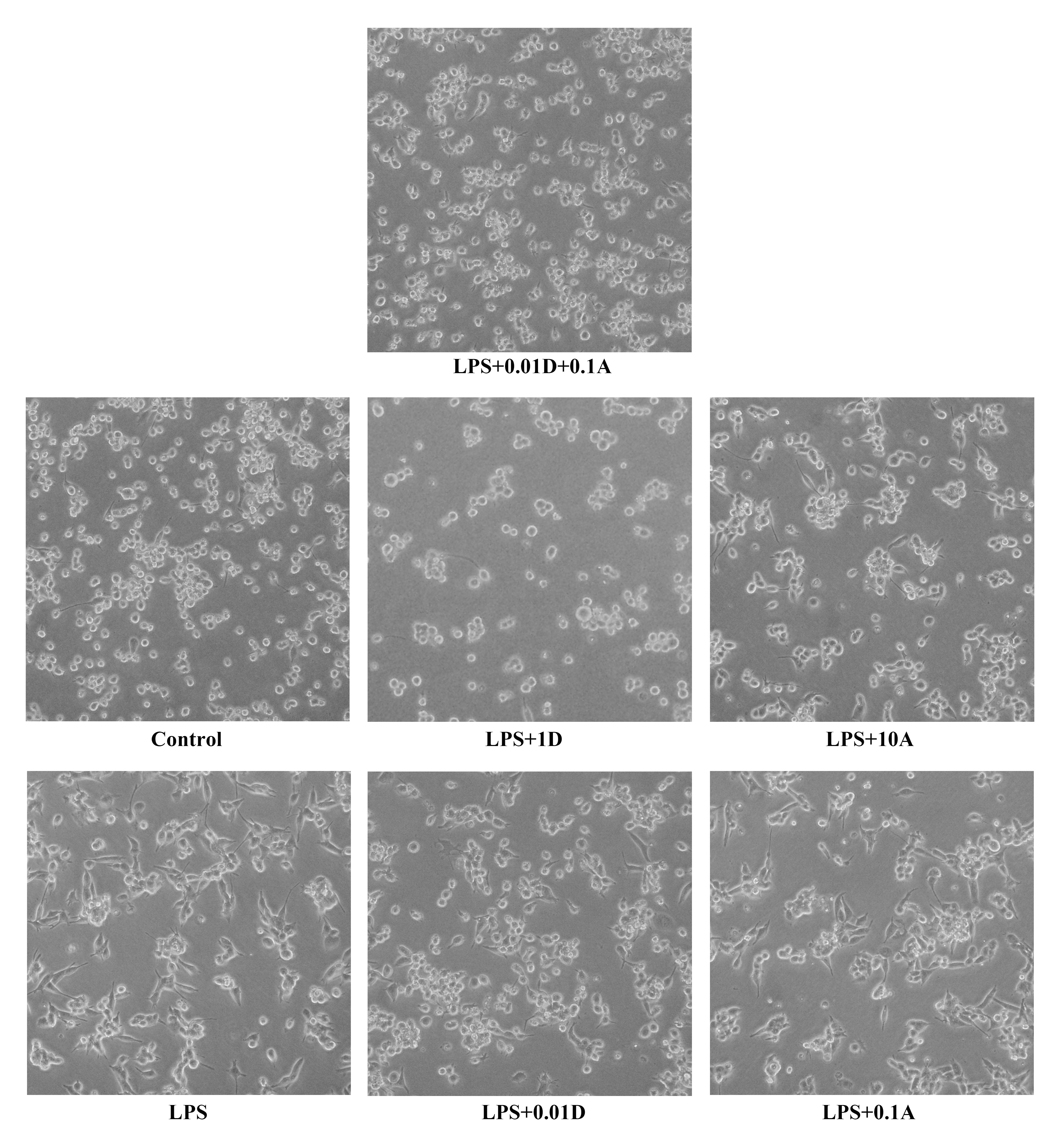

Supplement: Supplementary file 1 — Additional file 1: File S1. The inclusion and exclusion criteria. Table S1. RT-PCR Primers used in this study. Table S2. Baseline characteristics and outcome of CSDH patients treated with a combination regimen or ATO monotherapy. Table S3. Baseline characteristics and outcomes of conservatively treated patients who have good efficacy or switched to surgery. Table S4. The functions of these proteins identified, but not specifically discussed in the manuscript. Figure S1. o-ATO and p-ATO in CSDH patients. Figure S2. Concentrations of ATO and DEX in HUVEC. Figure S3. Effects of ATO and DEX on expression of drug transport and catabolism-related proteins in macrophages. Figure S4. Monocytes and macrophages in the haematoma of CSDH patients. Figure S5. The differentiation of THP-1 cells into macrophages stimulated by PMA. Figure S6. LPS can effectively simulate the effect of haematoma on THP-1 macrophages. Figure S7. Regulation of ATO and DEX on the morphological changes of THP-1 macrophages. Figure S8. The effect of ATO and DEX on the MFI of CD86 and CD163 in macrophages. Figure S9. The concentrations of ET-1 in the haematoma, serum and medium supernatant quantified by ELISA. [file 12974_2021_2257_MOESM1_ESM.zip › Fig. S7(non-updated).tif]

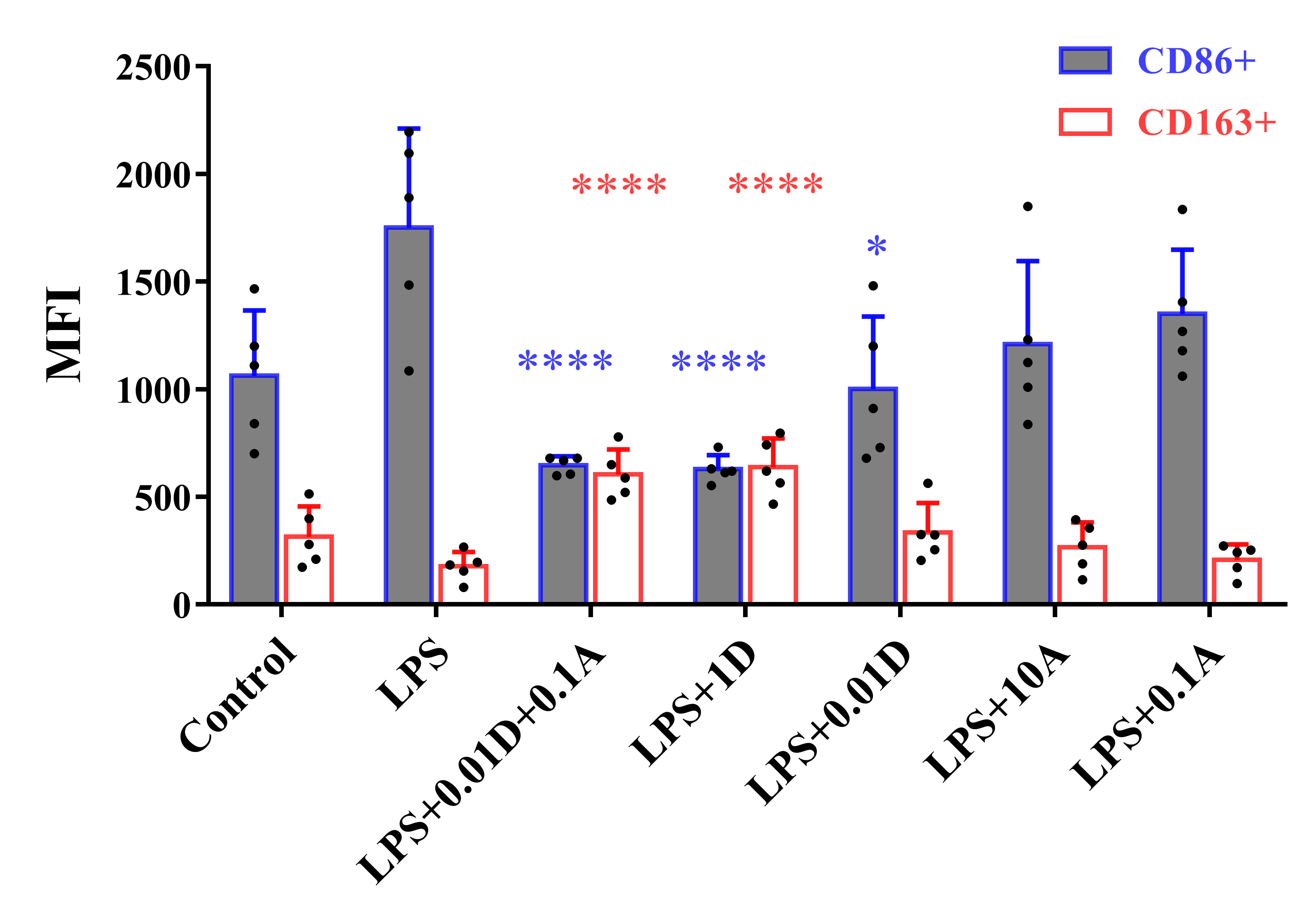

Supplement: Supplementary file 1 — Additional file 1: File S1. The inclusion and exclusion criteria. Table S1. RT-PCR Primers used in this study. Table S2. Baseline characteristics and outcome of CSDH patients treated with a combination regimen or ATO monotherapy. Table S3. Baseline characteristics and outcomes of conservatively treated patients who have good efficacy or switched to surgery. Table S4. The functions of these proteins identified, but not specifically discussed in the manuscript. Figure S1. o-ATO and p-ATO in CSDH patients. Figure S2. Concentrations of ATO and DEX in HUVEC. Figure S3. Effects of ATO and DEX on expression of drug transport and catabolism-related proteins in macrophages. Figure S4. Monocytes and macrophages in the haematoma of CSDH patients. Figure S5. The differentiation of THP-1 cells into macrophages stimulated by PMA. Figure S6. LPS can effectively simulate the effect of haematoma on THP-1 macrophages. Figure S7. Regulation of ATO and DEX on the morphological changes of THP-1 macrophages. Figure S8. The effect of ATO and DEX on the MFI of CD86 and CD163 in macrophages. Figure S9. The concentrations of ET-1 in the haematoma, serum and medium supernatant quantified by ELISA. [file 12974_2021_2257_MOESM1_ESM.zip › Fig. S8(updated).tif]

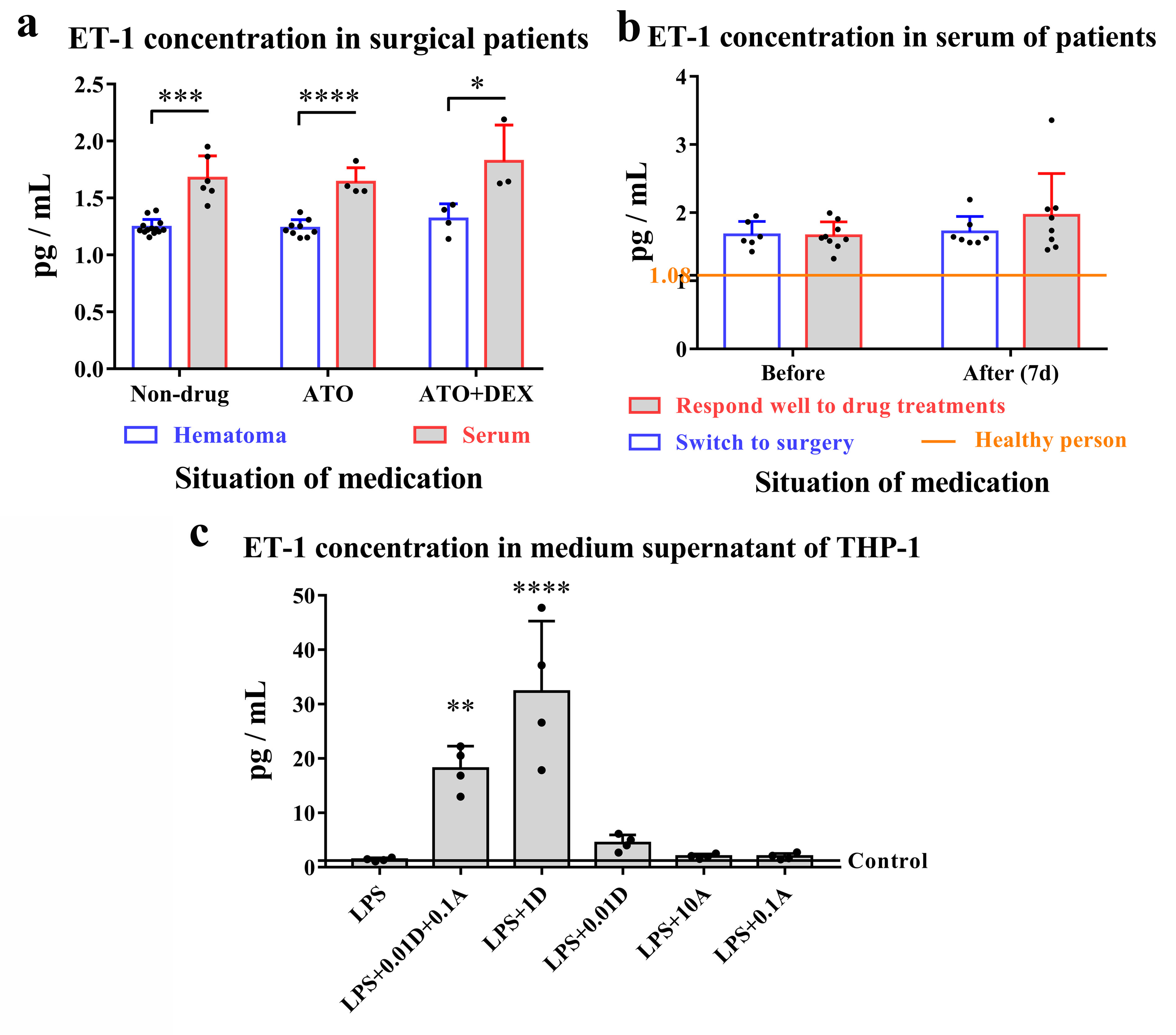

Supplement: Supplementary file 1 — Additional file 1: File S1. The inclusion and exclusion criteria. Table S1. RT-PCR Primers used in this study. Table S2. Baseline characteristics and outcome of CSDH patients treated with a combination regimen or ATO monotherapy. Table S3. Baseline characteristics and outcomes of conservatively treated patients who have good efficacy or switched to surgery. Table S4. The functions of these proteins identified, but not specifically discussed in the manuscript. Figure S1. o-ATO and p-ATO in CSDH patients. Figure S2. Concentrations of ATO and DEX in HUVEC. Figure S3. Effects of ATO and DEX on expression of drug transport and catabolism-related proteins in macrophages. Figure S4. Monocytes and macrophages in the haematoma of CSDH patients. Figure S5. The differentiation of THP-1 cells into macrophages stimulated by PMA. Figure S6. LPS can effectively simulate the effect of haematoma on THP-1 macrophages. Figure S7. Regulation of ATO and DEX on the morphological changes of THP-1 macrophages. Figure S8. The effect of ATO and DEX on the MFI of CD86 and CD163 in macrophages. Figure S9. The concentrations of ET-1 in the haematoma, serum and medium supernatant quantified by ELISA. [file 12974_2021_2257_MOESM1_ESM.zip › Fig. S9(updated).tif]
